# MicroRNAs Differentially Expressed in Postnatal Aortic Development Downregulate Elastin via 3′ UTR and Coding-Sequence Binding Sites

**DOI:** 10.1371/journal.pone.0016250

**Published:** 2011-01-31

**Authors:** Claus Eric Ott, Johannes Grünhagen, Marten Jäger, Daniel Horbelt, Simon Schwill, Klaus Kallenbach, Gao Guo, Thomas Manke, Petra Knaus, Stefan Mundlos, Peter N. Robinson

**Affiliations:** 1 Institute for Medical Genetics, Charité-Universitätsmedizin Berlin, Berlin, Germany; 2 Berlin-Brandenburg Center for Regenerative Therapies (BCRT), Charité-Universitätsmedizin Berlin, Berlin, Germany; 3 Institute for Biochemistry, Freie Universität Berlin, Berlin, Germany; 4 Department of Cardiac Surgery, University Hospital Heidelberg, Heidelberg, Germany; 5 Max Planck Institute for Molecular Genetics, Berlin, Germany; The University of Hong Kong, Hong Kong

## Abstract

Elastin production is characteristically turned off during the maturation of elastin-rich organs such as the aorta. MicroRNAs (miRNAs) are small regulatory RNAs that down-regulate target mRNAs by binding to miRNA regulatory elements (MREs) typically located in the 3′ UTR. Here we show a striking up-regulation of miR-29 and miR-15 family miRNAs during murine aortic development with commensurate down-regulation of targets including elastin and other extracellular matrix (ECM) genes. There were a total of 14 MREs for miR-29 in the coding sequences (CDS) and 3′ UTR of elastin, which was highly significant, and up to 22 miR-29 MREs were found in the CDS of multiple ECM genes including several collagens. This overrepresentation was conserved throughout mammalian evolution. Luciferase reporter assays showed synergistic effects of miR-29 and miR-15 family miRNAs on 3′ UTR and coding-sequence elastin constructs. Our results demonstrate that multiple miR-29 and miR-15 family MREs are characteristic for some ECM genes and suggest that miR-29 and miR-15 family miRNAs are involved in the down-regulation of elastin in the adult aorta.

## Introduction

Elastogenesis, one of the crucial components of the development of the aorta, is a complex process that encompasses transcriptional and posttranscriptional regulation as well as coordinated assembly of multiple molecules in the extracellular milieu. Elastin is the dominant ECM protein in the tunica media of the arterial and aortic wall, which is composed of a dense population of concentrically organized vascular smooth muscle cells (vSMCs) that synthesize elastin molecules and secrete them as soluble, hydrophobic monomers termed tropoelastin. Elastin is a major synthetic product of aortic tissues in early stages of postnatal development, but synthesis of the protein generally peaks early during arterial growth, decreases rapidly with further development, and essentially ceases in the aortic tissue of adults [1]. In humans, mature elastic fibers are extraordinarily stable, showing negligible turnover rates in many adult tissues including lung and aorta [2]–[4]. This low turnover is presumably a major factor in the loss of elasticity due to degradative changes in aging of connective tissues including the development of aortic stiffening [5].

Regulation of elastic-fiber formation in the aorta and other tissues is a complex and multistep process involving a number of transcription factors, elastin-binding protein, fibulin-4 and -5, and lysyl oxidase [6]–[9]. TGF-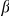-induced SMAD2 and SMAD3 signaling plays essential roles in the development of the aorta by promoting the expression of both vSMC-specific genes and of genes encoding components of the ECM of the aortic wall [10]. TGF-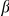 can induce both increases in elastin transcription [11] or can stabilize elastin mRNA [12]. The mechanisms of stabilization are not yet entirely clear but require the involvement of SMAD signaling, PKC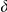, and p38 [13]. Destabilization of mRNA is an important contributing factor in the decline in production of aortic elastin taking place during normal postnatal growth [1].

MicroRNAs (miRNAs) represent a class of regulatory small RNAs that play a role in a large number of biological processes including differentiation and organ development [14]. Interaction of miRNAs with their targets primarily leads to negative regulation by means of inhibition of translation initiation or other forms of translational repression as well as by mRNA degradation [14].

Interaction of miRNAs with their targets primarily leads to negative regulation by means of inhibition of translation initiation [15] or other forms of translational repression [16], [17] as well as by mRNA degradation [18]. Although miRNAs have effects on both mRNA stability and translation, a substantial amount of the effects of miRNAs on protein synthesis can be explained by a reduction in mRNA abundance [19]–[22], which is strong enough to identify specific signatures of miRNAs on target mRNA expression computationally [14], [23]. The most important determinant of miRNA specificity is Watson-Crick pairing to the miRNA seed centered on nucleotides 2–7 of the miRNA. Although some exceptions are known, almost all validated miRNA binding sites are located in the 3′ UTR of target mRNAs [14]. Multiple sites have additive or cooperative effects, especially if located within about 40 nucleotides (nt), but no closer than 8 nt to one another [14]. Many miRNAs consist of families in which all the members share a common seed sequence, but vary in their sequence elsewhere. Because the seed region is the main determinant of specificity, it is believed that the members of a given miRNA family regulate a very similar set of target mRNAs [24].

Here, we perform expression profiling of mRNAs and miRNAs in the neonatal (*neo*) and adult (*w6*) aorta of C57BL/6 mice in order to gain a better understanding of the role of miRNA/mRNA networks in the developing aorta and in the characteristic postnatal shutting off of elastin gene expression. We found that members of two miRNA families play a prominent role in the shift in gene expression pattern in the aorta. Surprisingly, we found experimental and computational evidence that multiple miRNA binding sites in the coding sequence of the elastin gene as well as several other genes for proteins of the extracellular matrix (ECM) are involved in miRNA-mediated regulation of their expression.

## Results

### Identification of Differential Regulation of miR-15 and miR-29 family miRNAs and their Target mRNAs in Postnatal Aortic Development

**Figure 1:**
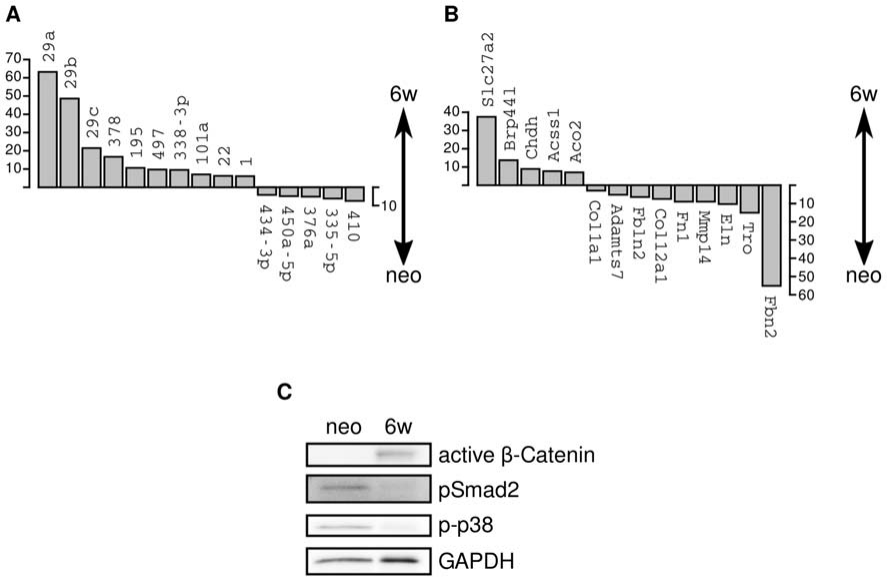
Shifts in mRNA and miRNA expression and TGF-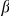 and Wnt pathway activity in postnatal aortic development. **A** The fold-change of the normalized expression levels of the most highly differentially regulated miRNAs as measured by microarray hybridization is displayed. **B** The fold-change of the normalized expression levels of the most highly differentially regulated mRNAs annotated to the GO terms *mitochondrion* (up-regulated at *w6*) and *extracellular matrix* (down-regulated at *w6*). (A,B) The fold-change between the *neo* and *w6* aortic samples is shown with the direction of the change being indicated by the arrows. Data are presented as mean of three measurements (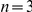). Each mRNA or miRNA had a Benjamini-Hochberg [68] corrected 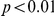 and an absolute fold change of at least 2. **C** Western Blot analysis with antibodies against active 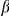-catenin, phosphorylated Smad2, and phosphorylated p38. TGF-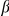 responsive Smad2 and p38 were activated in *neo* aorta and show reduced activity in *w6* specimens. By contrast, active (i.e. dephosphorylated) 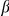-catenin was detectable in aortic samples from adult mice only. Equal loading was confirmed by GAPDH staining. Full scans are shown in Supplementary Fig. S2. Samples were run on the same gel.

In order to identify miRNAs involved in postnatal thoracic aortic development processes, we extracted the thoracic aorta from neonatal and six-week old C57BL/6 mice and compared the mRNA and miRNA expression profiles. Among the 650 miRNAs represented on the microarray, 54 were found to be significantly more highly expressed in the aortic samples of the six week old mice (“*w6*”) and 11 significantly more highly expressed in the samples from the neonatal (“*neo*”) mice (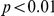 and foldchange 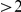; Fig. 1A and Supplementary Tables S1–S2 and Supplementary Fig. S1A). mRNA expression profiles were generated in parallel to the miRNA data. Numerous genes were found to be significantly differentially expressed. There were 2,142 genes with significantly higher expression in the *neo* samples, 78 genes of which displayed an at least two-fold higher expression in the *neo* than in the *w6* aortic samples with a corrected 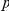-value 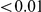. On the other hand there were 1,327 genes with significantly higher expression in the *w6* samples, 34 of which were expressed at an at least two-fold higher level in the *w6* than in the *neo* aortic samples with a corrected 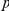-value 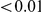 (Fig. 1B and Supplementary Tables S3–S4 and Supplementary Fig. S1B).

We performed Gene Ontology (GO) analysis on these genes using model-based gene set analysis (MGSA), which analyzes all GO terms at once by embedding them in a Bayesian network in order to provide high-level, summarized views of core biological processes [25]. The most highly ranked term for the genes with higher expression in the *neo* aortic samples was *extracellular matrix*. 74 of the total of 283 genes annotated to this term in the mouse genome were among the genes with higher expression in the neonatal aorta, including the genes for elastin, fibrillin-1 and -2, several metallopeptidases, and numerous collagens. On the other hand, the most highly ranked term for the genes with significantly higher expression in the *w6* aortic specimens was *mitochondrion* (Table 1). Since vSMCs, the major contractile cells in the aorta, require energy generation by oxidative phosphorylation to maintain blood pressure [26], the results of the GO analysis could possibly reflect higher energy needs of the adult aorta related to higher tensile stress on the adult aortic wall because of higher blood pressure and a larger aortic diameter.

**Table 1:** GO model-based gene set analysis (MGSA) [25] using the Ontologizer [65].

Significantly Higher Expression in Aortic Specimens of Neonatal Mice				
ID	Name	Marginal	Study Count	Population Count
GO:0031012	extracellular matrix	0.969	71	269
GO:0040029	regulation of gene expression, epigenetic	0.916	14	39
GO:0016055	Wnt receptor signaling pathway	0.821	30	129
GO:0017053	transcriptional repressor complex	0.506	7	16

Significantly Higher Expression in Aortic Specimens of 6-week old Mice				
ID	Name	Marginal	Study Count	Population Count
GO:0005739	mitochondrion	1.00	327	896
GO:0005811	lipid particle	0.964	6	11
GO:0016411	acylglycerol O-acyltransferase activity	0.810	6	11
GO:0019882	antigen processing and presentation	0.734	17	49
GO:0043353	enucleate erythrocyte differentiation	0.700	4	7
GO:0006084	acetyl-CoA metabolic process	0.541	23	32

For the analysis, 2,142 genes with significantly higher expression in the *neo* aortic samples and 1,327 genes with significantly higher expression in the *w6* aortic samples were compared to all 27,827 genes represented on the microarray. Study count and population count indicate the number of genes in the study sets of differentially regulated genes and the population set of all genes on the microarray that are annotated to the GO term in question. Terms with marginal probabilities higher than 0.5 are more likely than not to be related to the observed profile of differential expression [25].

An additional 30 of the genes with higher expression in the *neo* aortic samples were annotated to *Wnt receptor signaling pathway*, which was the term ranked in third place by MGSA. Although several of these genes such as *Fzd1* and *Bcl9* [27] are involved in the positive mediation of Wnt signaling, many of the genes typically have an inhibitory effect on Wnt signaling, including *Dkk2* and *Dkk3* [28], *Sfrp1*, *Sfrp2* and *Sfrp5* [29], *Dact1* [30], *Kremen1* [31], and *Tle1*, *Tle2*, and *Tle3* [32], suggesting the possibility of a negative regulatory program for Wnt signaling in the neonatal aorta. This observation prompted us to examine activated 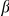-catenin, a marker of canonical Wnt activity, in the aorta by Western blotting of samples from which gene expression profiling had been performed. Given the known role of TGF-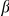 signaling in the development of the aorta [10], we additionally performed Western analysis of markers of TGF-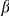 signaling activity as a control.

Western blotting with antibodies against the phosphorylated forms of SMAD2 and p38 confirmed clear activity of the SMAD-dependent as well as the SMAD-independent TGF-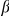 signaling pathway in the *neo* but not the *w6* aorta. In contrast, active (i.e. dephosphorylated) 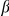-catenin was only detected in the *w6* aortic samples, indicating activity of the Wnt-signaling pathway in the adult but not in the neonatal aorta (Fig. 1C).

In summary, we found evidence of a shift in expression from genes involved in extracellular matrix (ECM) production to genes involved in oxidative energy production in the murine aorta during the first 6 weeks of life with a concomitant shift from activation of TGF-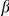 signaling to Wnt signaling.

### Differentially Expressed miRNAs are Significantly Correlated with the Expression Pattern of their Target mRNAs in the Postnatal Developing Aorta

**Figure 2:**
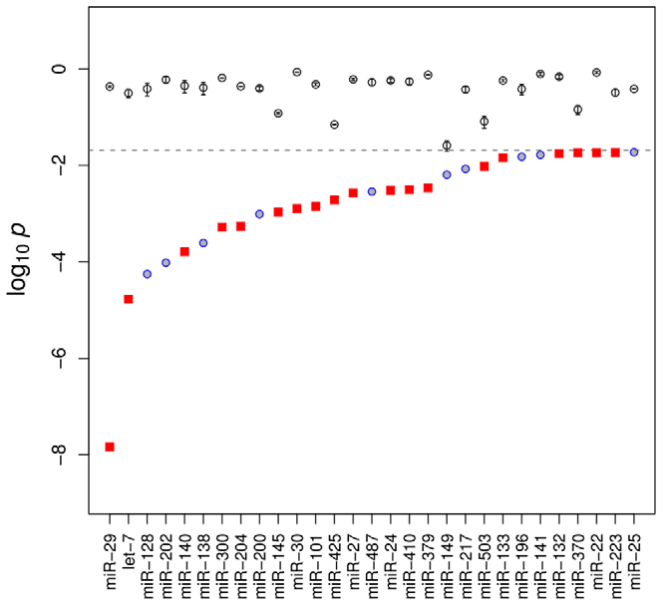
miRNA Signature Analysis of mRNA Gene Expression Data. miRNAs with significantly lower target-gene expression in the aortic samples from six-week old mice as determined by the Wilcoxon rank sum test [23]. The logarithm of the probabilities is shown as red squares (for miRNAs that themselves were found to be differentially expressed by microarray hybridization) or as blue circles (for miRNAs that were not differentially expressed). For instance, a value of 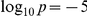 for a given miRNA would correspond to a probability of 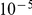 of observing the distribution of the corresponding miRNA seed sequences in the mRNA expression profile by chance. Additionally, the mean probability (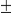 standard error) derived from ten random sets is plotted for each miRNA as an additional circle with error bars.

The gene targets of highly expressed miRNAs on aggregate tend to display a lower overall expression [23], [33], which can be detected in microarray datasets by comparing their expression levels of target vs. non-target mRNAs with a Wilcoxon rank-sum test [34]. Consistent with the identification of miR-29 family members as the most highly differentially regulated miRNAs in postnatal aortic development, the miR-29 family was found to have the most significant gene-expression signature in the mRNA expression profile of the *w6* samples (Fig. 2). 20 of the 30 miRNA families found to be significant on the Wilcoxon test were also shown to be significantly down-regulated experimentally (these miRNAs are shown as red boxes in Fig. 2). If we assume the probability of choosing a down-regulated miRNA family by chance is about 0.095 (54 of the 567 miRNA probes on the microarray were found to be significantly down-regulated), then we can estimate the probability of observing this degree of overlap by chance using the binomial distribution as 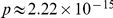. This suggests that the observed shift in the miRNA expression profile is related to the shifts in the mRNA expression profile in the developing aorta.

### 3′ UTR and Coding Sequence miRNA Response Elements Down-Regulate Elastin in Luciferase Reporter Assays

**Figure 3:**
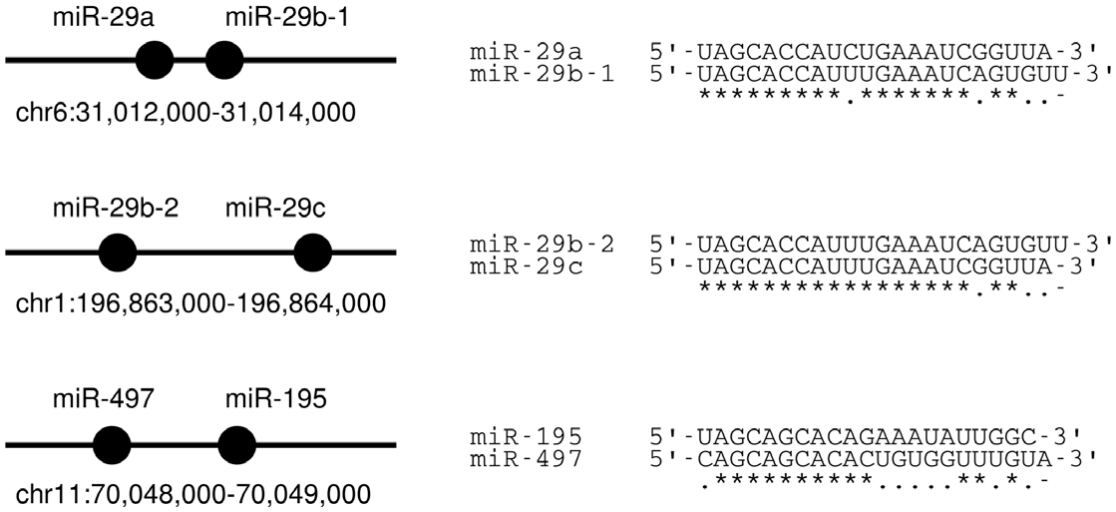
miR-29 and the miR-15 family members miR-195/miR-497 form three intergenic clusters in mouse chromosomes 1, 6, and 11. All three isoforms of miR-29 share the same seed sequence at nucleotides 1–8, corresponding to a sequence of UGGUGCUA in target mRNAs. miR-195 and miR-497 share the same seed sequence at nucleotides 2–8 but differ in position one.

Five of the six miRNAs that were most up-regulated in the *w6* aortas belonged to just two miRNA families, miR-29 and miR-15. An additional member of the miR-15 family, miR-15a, was up-regulated 4.1-fold in the *w6* aortic specimens (Supplementary Table S2). The seed sequences of miR-15 miRNAs (AGCAGCA) and miR-29 family miRNAs (AGCACCA) differ by only one nucleotide (Fig. 3). This motivated us to ask what role these miRNAs play in the regulation of *Eln*.

**Figure 4:**
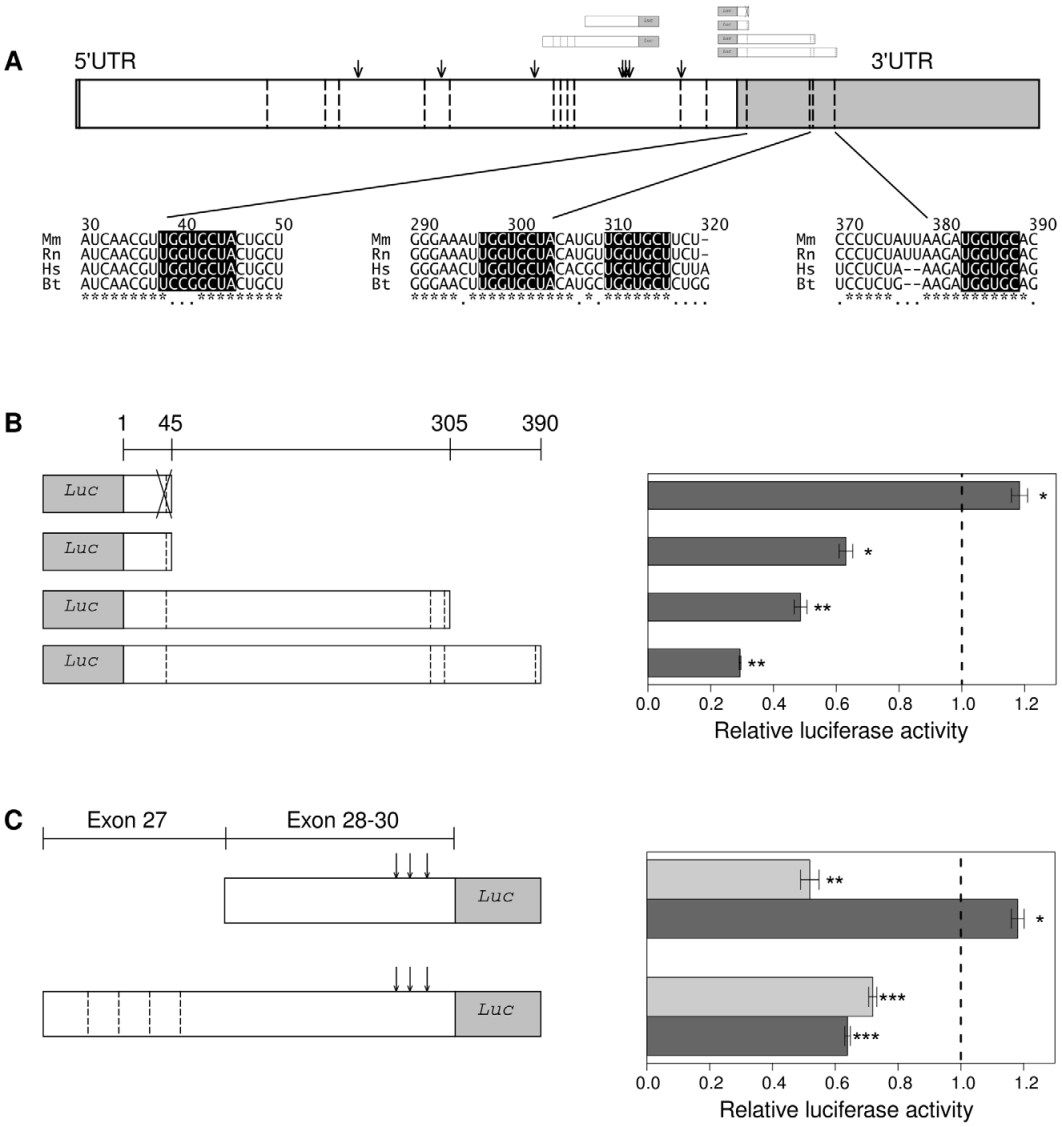
3′ UTR and CDS MREs for miR-29, miR-195, and miR-497 in the elastin gene. In the cartoons of the mouse elastin gene (A) and the luciferase constructs (B,C) miR-29 MREs (UGGUGCU) are indicated by dashed lines, and miR-15 MREs (UGCUGCU) by arrows. **A** The shaded sequences show the 3′ UTR MREs that are analyzed in B. Three MREs match the seed sequence perfectly, and a fourth MRE at position 380–388 of the *Eln* 3′ UTR matches positions 3–8 of all miR-29 family seed sequences but positions 9–11 only of miR-29a. (B,C) Dark gray bars represent treatment with miR-29 mimic, light gray bars treatment with miR-497 mimic. Data are presented as mean 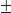 SE for triplicate analyses (* 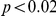, ** 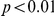, and *** 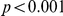). The luciferase activity of the empty vector cotransfected with miRNA precursors is displayed as vertical line at 1.0. **B** 3′ UTR reporter assays. On top a mutant construct is shown in which the miR-29 MRE is crossed out. Treatment of cells with miR-29a mimic reduced expression of all wildtype constructs but not of the mutant construct. Longer constructs displayed a greater degree of reduction. **C** CDS reporter assays. The construct on top with *Eln* exons 28 to 30 contains three miR-15 but no miR-29 MREs. The longer construct at the bottom with *Eln* exons 27 to 30 additionally contains four miR-29 MREs. According to the presence of MREs the short construct was repressed only by miR-497 mimics, but the long construct was repressed by both mimics. Each experiment was independently repeated at least twice with similar results.

Inspection of the 3′ UTR of *Eln* revealed four potential binding sites for the most highly up-regulated miRNA, miR-29 (Fig. 4A). We developed a series of constructs to investigate whether these sites can act synergistically to reduce expression of *Eln*. We chose a miR-29a precursor for these studies because all three isoforms of miR-29 share the same seed sequence (nucleotides 2–8; Fig. 3), and miR-29a showed the highest degree of differential expression in our experiments (Fig. 1A). As shown in Fig. 4B, cotransfection of miR-29a precursor with a 3′ UTR construct containing one miR-29 MRE resulted in a reduction in luciferase activity to about 60%. Mutation of this MRE abolished the activity of the miR-29a precursor. Longer constructs with two or three further miR-29 MREs in the *Eln* 3′ UTR displayed a higher degree of reduction in luciferase activity when exposed to miR-29a precursor. These results confirm previous observations that the 3′ UTR of murine *Eln* is a miR-29 target [35] and additionally show that the multiple miR-29 MREs act synergistically (Fig. 4B).

Computational analysis revealed eleven additional 7–8mer binding sites for miR-29 in the coding sequence (CDS) of elastin (Fig. 4A, dashed lines), as well as eight MREs with perfect complementarity to the seed sequence of the miR-15 family members miR-195/miR-497 (Fig. 4A, arrows). To investigate the functionality of the MREs in the CDS of *Eln*, we designed luciferase reporter constructs to contain segments of the *Eln* coding sequence followed by the entire coding sequence of the firefly luciferase gene, thereby creating a fusion protein (Fig. 4C). The first such construct contains three miR-497/miR-195 MREs but no miR-29 MREs, and showed repression by the miR-497 precursor but not by the miR-29 precursor. The second construct additionally contained four miR-29 MREs, and was significantly responsive to both miRNA precursors, indicating a specific effect of miR-15 and miR-29 MREs in the CDS of murine *Eln*. There are no MREs for miR-29 and miR-15 family miRNAs in the entire coding sequence of the firefly and renilla luciferase genes.

### Genes with multiple miR-15 and miR-29 family MREs in coding sequence and 3′ UTR are down-regulated in the *w6* aorta

These results motivated us to ask whether other genes with multiple miR-29 family MREs are also down-regulated in the *w6 aorta*. Computational sequence analysis identified 50 genes containing at least five MREs for miR-29 in the CDS and 3′ UTR sequences. Many of these genes also demonstrated multiple MREs for the miR-15 family (Supplementary Table S5). 17 of the genes in this list showed statistically significant differential expression in our experiments. 16 of these genes were down-regulated in the *w6* aorta. Since 58% of all differentially regulated genes were down-regulated (78 genes were down-regulated and 56 were up-regulated with a corrected 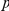-value 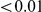), we can estimate the chance that 16 of 17 differentially regulated genes with at least 5 CDS and 3′ UTR MREs for miR-29 are down-regulated using the binomial distribution as 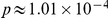.

**Figure 5:**
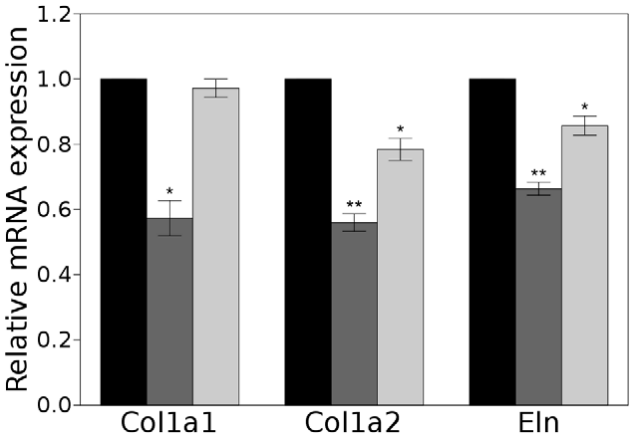
miRNAs targeting coding sequence MREs can reduce the expression level of ECM genes in RFL-6 cell culture. Triplicate qPCR analyses for *Col1a1*, *Col1a2*, and *Eln* following treatment of RFL-6 cells with scrambled (black), miR-29 (dark grey), or miR-195 (light grey) precursor. Data are presented as mean 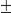 SEM for three independent treatments (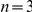, 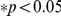, 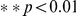).

We then proceeded to test the effect of treating RFL-6 cells with miR-29 or miR-195 mimics on the expression of *Eln*, *Col1a1*, and *Col1a2*. RFL-6 cells (which derive from the rat) are known to produce abundant amounts of elastin mRNA [36] and were therefore chosen for the experiment. In the rat, *Col1a1* has 20 miR-29 MREs in its CDS and one in its 3′ UTR, and has 4 MREs for miR-15 family miRNAs in its CDS and one in its 3′ UTR; *Col1a2* has 15 miR-29 MREs in its CDS and none in its 3′ UTR, and 7 miR-15 family MREs in its CDS; and *Eln* has 10 miR-29 MREs in its CDS and 3 in its 3′ UTR, and 9 MREs for miR-15 and none in the 3′ UTR. Mimics for miR-29 or miR-195 led to repression of elastin expression as measured by qPCR (Fig. 5), and treatment of the cells with antagonists of miR-29 had the opposite effect (Supplementary Fig. S3). Although a secondary effect of miR-195 cannot be excluded, this result is consistent with biologically active MREs for miR-195 in the CDS of the elastin gene. In addition, *Col1a2* has neither miR-15 nor miR-29 MREs in its 3′ UTR and was responsive to both miR mimics. Although *Col1a1* has one miR-15 and one miR-29 MRE in its 3′ UTR, the great majority of MREs for these miRNAs are located in its CDS. Thus, we conclude that the posttranscriptional repression of elastin in the adult aorta is cooperatively mediated by five of the top six up-regulated miRNAs, that multiple MREs in the CDS of the elastin gene contribute to the regulation, and that similar regulatory mechanisms may apply to *Col1a1* and *Col1a2*.

### MREs for miR-15 and miR-29 are highly overrepresented in the mRNA sequence of *Eln*

**Figure 6:**
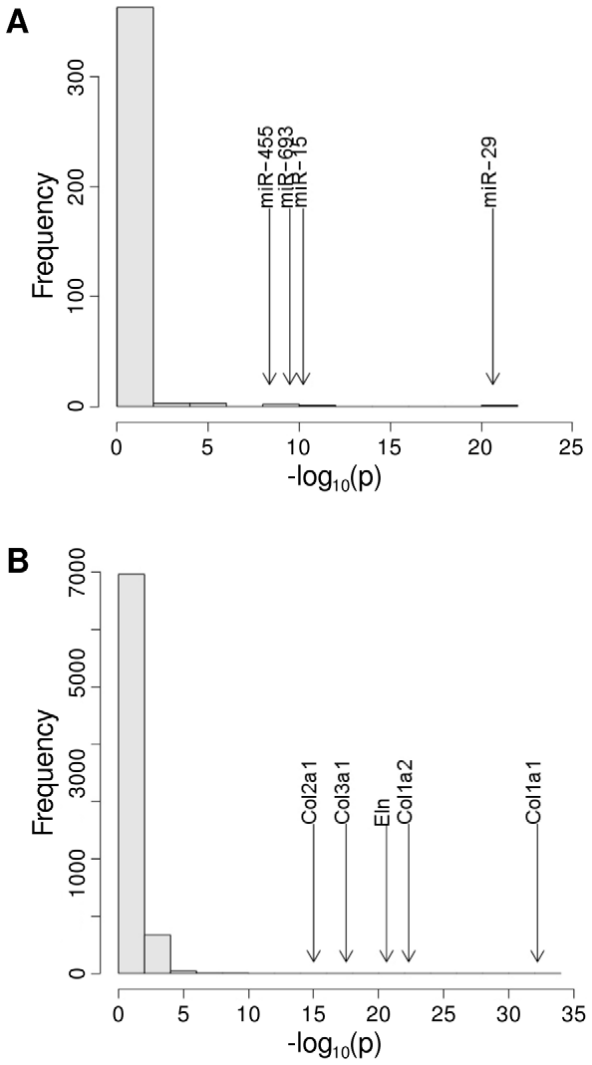
miR-29 is overrepresented in *Eln* and multiple collagen genes. **A** When compared to the expectation under a random sequence model, *Eln* mRNA possesses a highly significant excess of miR-29 and miR-15 MREs. The number of MREs for each of 373 miRNA families in the entire elastin mRNA sequence was counted and compared to the expectation under a random sequence model based on the Poisson distribution. Results are displayed as a histogram of the negative decadic logarithm (for instance, the 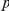-value for miR-29 of 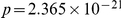 is displayed as 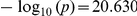). Arrows indicate the position of the four most highly significant miRNAs. **B** The number of MREs for miR-29 was counted in the mRNA sequences of each of 33,394 murine genes, using only the longest transcript for genes with alternative transcripts. The counts of miR-29 MREs were compared to a random sequence model. Results for the 7,675 genes with at least one MRE for miR-29 are displayed as a histogram of the negative decadic logarithm. Most mRNAs did not show significant enrichment. In addition to *Eln*, a number of collagen genes showed highly significant enrichment. See also Supplementary Table S5 for counts of miR-15 and miR-29 family MREs.

We developed a simple statistical test for overrepresentation of MREs in mRNA sequences (see methods) and used it to analyze the counts of MREs for 373 miRNA families in the entire mRNA sequence of *Eln*. miR-29 showed the most highly significant enrichment (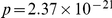), and MREs for the miR-15 family showed the second most significant enrichment (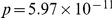). Most miRNAs did not show significant enrichment, and only four displayed a 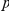 value less than 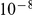 (Fig. 6A). We then performed a similar analysis on all murine mRNA sequences. There were 7,675 mRNA sequences with at least one MRE for miR-29. There were a total of five genes with 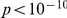 for enrichment in MREs for miR-29. Intriguingly, all five of these genes have prominent roles in the extracellular matrix, including the *Eln* gene and four collagen genes (Fig. 6B).

A similar analysis was carried out on several other mammalian genomes for which complete sequences for the elastin mRNA could be identified. miR-29 MREs are consistently overrepresented in mRNA sequences for elastin, and miR-15 family MREs are overrepresented in rat, cow, and dog but to a lesser degree in humans, suggesting species-specific differences (Supplementary Table S6).

## Discussion

Here we have shown that miR-29 is an important regulator of elastin expression by means of synergistic MREs in its 3′ UTR as well as additional MREs in its CDS. To date, research on miRNA interactions in animals has been nearly exclusively confined to the 3′ UTR of the mRNA targets [37], and it has been suggested that active translation impedes miRNA-programmed RISC association with target mRNAs [38]. The assumption that miRNAs bind exclusively to the 3′ UTR of their target mRNAs has meant that miRNA databases such as TargetScan [39], [40], PicTar [41], and miRBase [42] currently only show predictions for the 3′ UTR of targets.

However, MREs in the CDS of a number of genes have recently been demonstrated to be active by in vitro reporter assays [43]–[46]. A recent work has suggested that about 20% of all RISC-mRNA binding events involve the CDS [47]. The binding of multiple miRNAs to a single target mRNA can increase the effectiveness of repression [40]. The finding of a total of 14 MREs for miR-29 as well as 13 for the miR-15 miRNA miR-195 in the coding and 3′ UTR sequence of *Eln* is highly statistically significant (Fig. 6), and to our knowledge a similar finding has not been previously reported for any miRNA.

The high multiplicity of MREs for miR-15 and miR-29 family miRNAs is reminiscent of the VGVAPG repeating peptide in elastin, which can induce macrophage chemotaxis and other biological responses by interaction with the elastin-binding protein [48]–[50]. The core motif GxxPG is present 28 times in the sequence of human elastin, and from 3 to 11 times in a number of matrix proteins including fibrillin-1, five collagens, and two tenascins [51]. The high multiplicity of MREs for miR-15 and miR-29 family miRNAs could be a reason for the relatively effective repression observed for miR-29 and miR-497 on the CDS constructs investigated in this study (Fig. 4C) as well as the effectiveness of miR-mimics in cell culture (Fig. 5).

**Figure 7:**
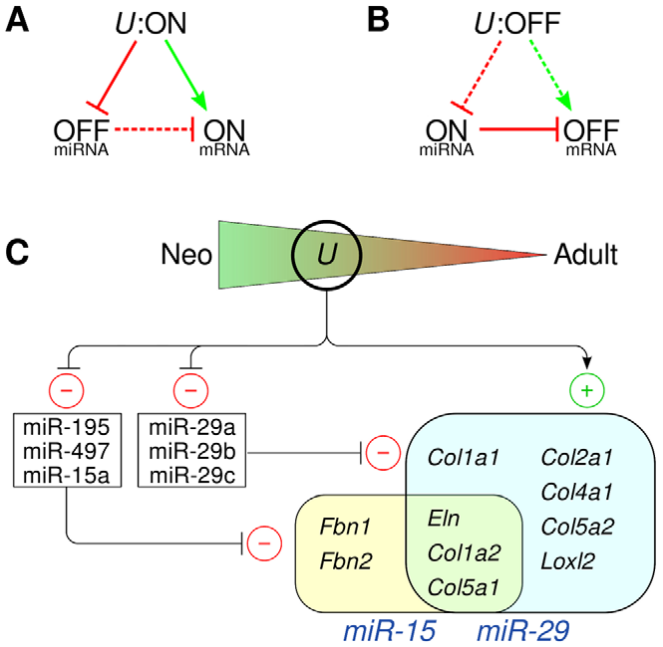
Putative model for a feed-forward miRNA/mRNA network controlling expression of key ECM genes in the developing aorta. *U* denotes *upstream* factors which tend to up-regulate ECM genes in the neonatal aorta and to suppress miR-29 and miR-15 family microRNAs. TGF-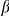 and Wnt signaling could be among the upstream factors (see text). **A** Neonatal aorta. If upstream factors are active (“U:*ON*”), they repress the expression of the miRNAs and induce the expression of numerous ECM genes such as *Eln*. **B** Adult aorta. If the upstream factors are inactive (“U:*OFF*”), there is less induction of the ECM genes. The miRNAs are no longer repressed, so that their expression levels increase and thereby provide an additional element of repression for the expression of the target mRNAs. In (A) and (B), green arrows indicate positive regulation, and red lines with bars negative regulation. Solid lines indicate that the regulatory reaction is active, and dashed lines indicate that it is inactive. **C** Putative model of miR-15/miR-29 and mRNA target gene networks. In the adult aorta, ECM genes tend to be down-regulated and the miRNAs to be up-regulated. Ten significantly down-regulated ECM genes are shown. Eight of them have at least five MREs for miR-29, five have at least five MREs for miR-15, including three genes that additionally have at least five MREs for miR-29.

Van Rooij and coworkers proposed a network feedback motif involving collagen, TGF-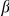 and miR-29 for the pathogenesis of cardiac fibrosis following myocardial infarction [35], [52]. Our results suggest the existence of a related miRNA/mRNA network that could be involved in the shift of the metabolic program of the aorta from processes involved in development and extracellular matrix (ECM) protein synthesis in the *neo* samples to oxidative energy metabolism in the *w6* samples, whereby there is a coordinate up-regulation of miR-29 and miR-15 family miRNAs and down-regulation of their ECM target genes including, prominently, elastin (Fig. 7). We note that the involvement of miR-15 miRNAs in the elastin feedback loop was not predicted in previous studies [35], [52], and indeed could not have been anticipated based on predictions of miRNA binding sites in which only the 3′ UTR is considered.

A limitation of our study is that a role for the miR-29 and miR-15 family MREs in the CDS of elastin and other matrix genes was not demonstrated in an *in vivo* model system. These microRNAs have been intensively studied in the context of myocardial infarction, where miR-29 was shown to act as a regulator of cardiac fibrosis in vivo [35]. However, such experiments are not able to distinguish between 3′ UTR and CDS binding events. Additionally, cardiac overexpression of miR-195 results in pathological cardiac growth and heart failure in transgenic mice [53], so that modulation of miR-15 miRNAs during development and maturation are unlikely to allow specific conclusions about the effect of miR-15 miRNAs on elastin metabolism. A second limitation is that our results are not able to fully clarify the nature of the upstream factors that up-regulate the expression of the ECM genes and down-regulate the expression of miR-29 and miR-15 family members. However, we note that TGF-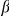 was previously shown to reduce miR-29 activity in cardiac fibroblasts [35], and Wnt signaling was shown to induce miR-29 in an osteoblastic cell line [54]. Our gene profiling and Western blot analysis suggest a shift from TGF-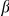 to Wnt signaling in the course of postnatal aortic development (Fig. 1C). Additionally, a number of ECM genes found to be down-regulated in aortic maturation in this study are both predicted or validated targets of miR-29 and also are transcriptionally regulated by TGF-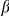, including *Col1a1* [55], *Eln* [56], *Col4a1* [57], and *Col6a3* [58]. Therefore, we postulate that TGF-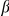 and Wnt signaling could be among the upstream factors (the “U” in Fig. 7) responsible for the shifts in miRNA and mRNA expression.

The network investigated in this work and shown in Fig. 7 is likely to be just one of several miRNA networks that modulate the process of aortic development and maturation. In fact, prominent roles of miR-145 in smooth-muscle cell fate and plasticity and smooth muscle cell maintenance and vascular homeostasis have recently been reported [59], [60]. Our data showed that miR-145 is significantly up-regulated during postnatal aortic development (Supplementary Table S2) and that there is a significantly lower target-gene expression in the aortic samples from six-week old mice [34] (Fig. 2). miR-23b cluster miRNAs target several SMAD genes, and were recently shown to be expressed at low levels during hepatic stem-cell differentiation, which in turn allows TGF-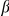 signaling and bile duct formation [61]. Our data also documented a statistically significant up-regulation of miR-23b cluster miRNAs (miR-23b, miR-27b, and miR-24) during postnatal aortic development (Supplementary Table S2), consistent with an inhibitory effect of these miRNAs on TGF-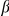 signaling. As with miR-29 and miR-145, computational analysis of the target gene profile for miR-27 and miR-24 showed a significant shift in the aortic samples from six-week old mice (Fig. 2), suggesting that these miRNAs also contribute to the mRNA expression profile in the adult aorta. A further differentially expressed miRNA whose target mRNA profile was not statistically significant, miR-378, is known to promote angiogenesis [62].

In conclusion, we have shown that miR-29 and the miR-15 family members miR-195 and miR-497 are differentially regulated between the newborn and the six-week old murine aorta, and using *in vitro* assays we have demonstrated that they regulate elastin by means of multiple MREs in both the 3′ UTR and the CDS. There is a highly significant overrepresentation of MREs for miR-29 in the mRNA sequences of the elastin and type 1 collagen genes throughout mammalian evolution. We suggest that the high expression of miR-29 and the miR-15 family member in the adult aorta may be an important factor for the physiological suppression of the production of elastin in the adult organism.

## Materials and Methods

### Sample Preparation, RNA isolation, and Microarray hybridization

Aortic samples ranging from the aortic valve to the diaphragm were harvested from 15 newborn (i.e. 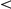
24 hours) and 15 six-week old C57BL/6 wildtype mice. Samples were rinsed in phosphate-buffered saline solution (PBS) to remove blood, and shock-frozen in liquid nitrogen. Pools of five frozen samples were pulverized and directly dissolved in RNAPure (PeqLab) to get three pooled aortic samples (n = 5) for each group. Total RNA was isolated using phenol/chloroform extraction. RNA integrity was confirmed using the Agilent RNA 6000 Nano Kit, and small RNA content was measured using the Agilent small RNA Kit and the Agilent 2100 bioanalyzer according to the manufacturer's instructions. For gene-expression analyses, 500 ng total RNA of each RNA sample was labeled using the Agilent single-color Quick-Amp Labeling Kit and hybridized on Agilent Whole Mouse Genome Microarrays (4×44K) according to the manufacturer's instructions. For analysis of microRNAs, 200 ng total RNA of each sample was labeled using the Agilent miRNA Labeling Reagent and Hyb Kit, and hybridized on Agilent Mouse miRNA Microarrays (8×15K, Part number G4472A, Sanger version 10.1, with 567 targeted miRNAs) according to the manufacturer's instructions.

Killing of all animals was conducted following national regulations for the care and use of laboratory animals and approved by the local legal representative (Landesamt für Gesundheit und Soziales Berlin: T 0438/08).

### Gene expression analysis by quantitative RT-PCR

For quantitative RT-PCR (Q-PCR), 1
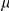
g total RNA from murine aortic samples was transcribed into cDNA (RevertAid H minus cDNA Synthesis Kit, Fermentas). Real-time PCR was performed on the Applied Biosystems 7900HT real-time PCR system in a total volume of 12
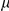
l in each well containing 6
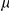
l of Power SYBR Green PCR Master Mix (Applied Biosystems), 5
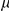
l cDNA (in a 1∶50 dilution) and 1
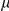
l primers (0.2
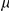
mol each) using standard PCR conditions. Samples were run in triplicates. *Actb* and *Gapdh* were used as endogenous controls.

### MicroRNA expression analysis by quantitative RT-PCR

MicroRNA TaqMan real-time PCR kits were purchased from Applied Biosystems. The reverse transcription was performed for all target microRNAs and the references U6 RNA and snoRNA202 using the TaqMan MicroRNA Reverse Transcription Kit. The reverse transcription products were used for the TaqMan real-time PCR reaction with TaqMan Universal PCR Master Mix, primers and TaqMan probes as provided with the miRNA TaqMan real-time PCR kits, and run in triplicates. Real-time PCR was performed on the Applied Biosystems 7900HT real-time PCR system. The PCR conditions were: 95°C for 10 min, and then 15 s at 95°C and 1 min at 60°C for 45 cycles. U6 RNA and snoRNA202 were used as endogenous controls.

### Reporter constructs

The 3′ UTR reporters were generated by cloning 3′ UTR fragments of *Eln* (ENSMUST00000015138) into the *Spe*I and *Hind*III sites of the pMIR-REPORT vector (Ambion). The first construct contains nucleotides 1–45 of the *Eln* 3′ UTR with a mutation of the miR-29 MRE sequence from UUGGUGCU to AACCACGA. The second construct contains nucleotides 1–45 of the *Eln* 3′ UTR with the wildtype sequence. The third construct contains nucleotides 1–305, and the fourth construct contains nucleotides 1–390 of the *Eln* 3′ UTR wildtype sequence. To generate coding sequence (CDS) reporter constructs, luciferase cDNA was amplified from pMIR-REPORT vector omitting the start codon and introducing a 5′ multiple cloning site containing (from 5′ to 3′) *Hind*III, *Not*I, *Sac*II, and *Kpn*I. The resulting construct was then cloned into pCDNA3 (Invitrogen) using *Hind*III and *Age*I. The CDS fragments of *Eln* (ENSMUST00000015138) containing a Kozak sequence followed by ATG in frame with luciferase were then cloned into *Hind*III and *Kpn*I sites of the modified pCDNA3 vector. Primers used to create the reporter constructs are given in Table 2. The position of the primers Eln-e27-F, Eln-e28-F, and Eln-e30-R refer to the corresponding exons of the mouse elastin transcript (ENSMUST00000015138).

**Table 2:** Sequences of the primers used for reporter constructs.

*Eln* 3′ UTR fragments and mutagenesis			
Name	Overhang	Restriction site	Specific sequence
Eln-Fra1-F	GACTG	ACTAGT	GGCTGCTTTGGGAAATCCTGT
Eln-Fra1-R	GACTG	AAGCTT	**TAGCACCAA** CGTTGATGAGG
Eln-Fra1mut-R	GACTG	AAGCTT	**CTCGTGGTT** CGTTGATGAGG TCGCGAGTCAGG
Eln-Fra2-R	GACTG	AAGCTT	AAGCACCAACATGTAGCACC
Eln-Fra3-R	GACTG	AAGCTT	GTGCACCATCTTAATAGAGGG

*Eln* CDS fragments			
Name	Overhang	Restriction site	Specific sequence
Eln-e27-F	AGCT	AAGCTT	ACCATG GCTGCCGCTGGGCTTGGAGCT
Eln-e28-F	AGCT	AAGCTT	ACCATG CCTGGATCGCTGGCTGCAT
Eln-e30-R	GTAC	GGTACC	ATACTGGGCAGCCTTAGCAG

Bold letters in the sequence of the reverse primers Eln-Fra1-R and Eln-Framut-R display the nucleotides subjected to mutagenesis.

### Luciferase Assay

The pMIR-REPORT vector (Ambion) containing reporter constructs was cotransfected into HEK-293 cells (institutional stock) with renilla luciferase control vector for normalization of the transfection efficiency, Pre-miR synthetic microRNA precursors or a scrambled miRNA negative control (Ambion) using Lipofectamine 2000 (Invitrogen) according to the manufacturer's protocol for cotransfection of DNA and siRNA. Cells were harvested 24 h after transfection, and the renilla and firefly luciferase activities in the cellular lysate were assayed by using the Dual-Glo Luciferase Assay (Promega) according to the manufacturer's protocol. Light intensity for each sample was measured by using a 96-well plate reader (Microlumat Plus LB96V Luminometer, Berthold), and each value from firefly luciferase construct was normalized by renilla luciferase.

### Modulation of microRNA activity in cell culture

RFL-6 is a rat fetal lung fibroblast cell line that expresses elastin abundantly and deposits elastic fibers [9], [36], [63]. RFL-6 cells were transfected with Pre-miR miRNA Precursor or Anti-miR miRNA Inhibitor (Ambion) using Lipofectamine 2000 (Invitrogen) according to the manufacturer's protocol to check the effects on mRNA expression. The number of MREs for miR-29 and miR-15 family miRNAs in the rat genes is based on the sequences NM_012722 (*Eln*), NM_053304 (*Col1a1*), and NM_053356 (*Col1a2*).

### Western blotting

Aortic samples were collected and shock-frozen. After mechanical homogenization cells were resuspended in lysis buffer (20 mM Tris, pH 7.4, 150 mM NaCl, 1% TritonX-100, 1 mM EDTA) containing protease and phosphatase inhibitors (Roche Applied Science, Mannheim, Germany) and lysed for 1 h at 4°C. Debris was pelletized by centrifugation, and protein concentrations were determined using the BCA method. 50 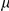
g of total protein were loaded per well. Proteins were detected on Western blots using anti-pSMAD2 antibodies (Zymed Laboratories/Invitrogen, Karlsruhe, Germany), anti-active-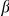-catenin (Millipore, Temecula, USA), anti-active p38 (Promega, Madison, USA). Equal loading was confirmed by anti-GAPDH antibodies (Cell Signaling/New England Biolabs, Frankfurt am Main, Germany). Chemiluminescence detection was performed using FemtoGlo reagents (P.J.K, Kleinbittersdorf, Germany) in a ChemiSmart5000 digital imaging system (Vilber- Lourmat/Peqlab, Erlangen, Germany). pSmad2, p-p38, and GAPDH antibodies were incubated simultaneously to avoid membrane stripping. The membrane was cut at about 70 kDa to allow separate incubation with anti-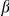-catenin antibodies.

### Computational analysis

#### Analysis of microarray data

After scanning the microarrays, the Agilent Feature Extraction Software v.10.5.1.1 (Agilent Technologies UK) was used with protocol GE1_105_Dec08 for gene expression, or with protocol miRNA_105_Dec08 for miRNA analyses to perform data extraction and quality control. The microarray data presented here are available in the ArrayExpress database [64] (www.ebi.ac.uk/arrayexpress) under the accession number E-MEXP-2342.

Data were normalized by setting the threshold of all values to 1. The median shift was normalized to the 75 percentile, and the baseline was transformed using the median of all samples. A subset of genes for data interrogation was generated that excluded probes that were absent or marginal in all of the six samples. Relative expression of each probe in aortic samples of newborn versus six-week old mice was determined. A *t*-test was performed followed by Benjamini and Hochberg multiple-testing correction. Gene-Ontology analysis was performed separately on the set of genes with significantly higher expression in the *neo* aortic samples and those with significantly higher expression in the *w6* samples by model-based gene set analysis [25] using the Ontologizer [65].

#### Analysis of miRNA signatures in mRNA expression profiles

Individual mRNA expression profiles were analyzed to reveal the effects of specific miRNAs using rank-sum tests [34]. We applied this approach both for the mean expression values of aortic samples from neonatal and six-week old mice as well as for the differences between the two datasets. If multiple probes were present for an mRNA gene, the mean value of all probes was used. The analysis was done using predicted miRNA targets taken from TargetScan [66].

#### miR-15 and miR-29 Overrepresentation in Eln

We can estimate the probability of observing at least 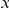 MREs for a specific miRNA in the mRNA sequence of a gene using the Poisson distribution. The null model is then the probability that the 7-nucleotide MRE starts at any given position of the mRNA sequence only by chance. If we assume equal nucleotide distribution, this is then 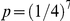. In the results reported in this manuscript, the null distribution was based on the exact nucleotide distribution calculated over all analyzed mRNA sequences, (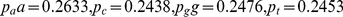). For miR-29, whose genomic MRE sequence is TGGTGCT, the probability is thus 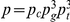, and the probability of an MRE starting at any given position can be modeled as a Bernoulli process with probability 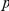.

If the length of an mRNA sequence is 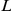, then there are 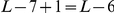 positions in the mRNA sequence at which the MRE could begin, if we disregard the mutual dependence of occurrences of MREs at positions 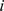 and 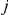, where 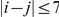, which is a reasonable simplification given the relative rarity of 7-nucleotide MREs. Then the probability of seeing 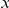 occurrences of an MRE in the sequence is given by the Poisson distribution with the parameter 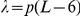: 
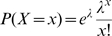
(1)


The probability of seeing at least 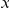 occurrences of an MRE is then 
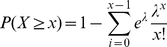
(2)


The rna.gbk file was downloaded from the NCBI Genome [67] ftp site on April 12, 2010, and one unique transcript was extracted for each gene by choosing the transcript with the longest total sequence length, resulting in 33394 unique genes. The file miR_Family_Info.txt was downloaded from the TargetScan website on April 12, 2010 [66], and was used to extract the mature miRNA sequences for 373 miRNA families. The number of 7-nucleotide MREs in the Eln mRNA sequence was calculated for each of these 373 miRNAs and the probability of seeing at least that many miRNAs was calculated according the Poisson distribution. Likewise, the number of 7-nucleotide miR-29 MREs was calculated for each of the 33,394 genes. There were 7675 mRNA sequences with at least one MRE for miR-29.

## Supporting Information

Figure S1Validation of differential expression of selected miRNAs (A) and mRNAs (B) by qPCR. The fold change of expression in the aortic samples of six-week old mice compared to that of neonatal mice is shown. Data are presented as mean 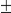 SEM (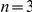). Data was normalized to one RNA sample of the neonatal mice, i.e. 1.0. The mean expression of U6 RNA and snoRNA202 served as endogenous control in microRNA analyses, and *Actb* as control for gene expression analyses. 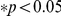, 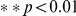.(PDF)

Figure S2 Full scans of images of the Western blots presented in the paper (Fig. 1C). The lower panel shows an enhanced exposure of the upper image.
(PDF)

Figure S3 Treatment with Anti-miR inhibitor against miR-29a can enhance the expression level of ECM genes in RFL-6 cell culture. Q-PCR for *Col1a1*, *Col1a2*, and *Eln* following treatment of RFL-6 cells with anti-miR-29 (dark grey) compared to untreated control (black). Error bars indicate standard error of triplicate qPCR analysis. The mean expression of *Actb* and *Gapdh* served as endogenous control.
(PDF)

Table S1microRNAs with significantly higher expression in aortic samples from neonatal mice. Data were normalized by setting the threshold of all values to 1. The median shift was normalized to the 75 percentile, and the baseline was transformed using the median of all samples. A subset of genes for data interrogation was generated that excluded probes that were absent or marginal in all of the six samples. Relative expression of each probe in aortic samples of newborn versus six-week old mice was determined. A *t*-test was performed followed by Benjamini and Hochberg (BH) multiple-testing correction[68]. There were 11 microRNAs with BH-corrected values 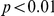, foldchange 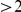, and normalized mean intensity 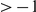. An additional six microRNAs were significant at 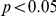.(PDF)

Table S2microRNAs with significantly higher expression in aortic samples from six-week old mice. Data analysis was performed as in Tab. S1. There were 54 microRNAs with BH-corrected values 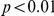, foldchange 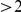, and normalized mean intensity 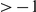. An additional 17 microRNAs were significant at 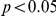.(PDF)

Table S3Genes with significantly higher expression in aortic samples from neonatal mice. Data were normalized by setting the threshold of all values to 1. The median shift was normalized to the 75 percentile, and the baseline was transformed using the median of all samples. A subset of genes for data interrogation was generated that excluded probes that were absent or marginal in all of the six samples. Relative expression of each probe in aortic samples of newborn versus six-week old mice was determined. A *t*-test was performed followed by Benjamini and Hochberg multiple-testing correction[68]. There were 82 probes corresponding to 78 genes with BH-corrected values 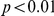 and foldchange 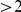. An additional 2,064 genes were significant at 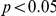 (Thus, there were a total of 2,338 probes and a total of 2,142 distinct significantly downregulated genes). Genes annotated to the GO term *extracellular matrix* are shown in bold.(PDF)

Table S4Genes with significantly higher expression in aortic samples from six-week old mice. Data analysis was performed as in Tab. S3. There were 57 probes corresponding to 56 genes with BH-corrected values 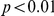 and foldchange 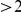. An additional 1,271 genes were significant at 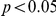 (Thus, there were a total of 1,509 probes and 1,327 sigificantly upregulated genes). Genes annotated to the GO term *mitochondrion* are shown in bold.(PDF)

Table S5There are 50 genes containing at least five MREs for miR-29 in the CDS plus 3′ UTR. MREs were identified using the definitions of 7–8 mer canonical miR seed-matches. As shown in Fig. 3 of the main manuscript, the first nucleotide of the seed sequences of miR-195 and miR-497 is different (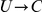). Therefore, some genes have a lower number of miR-497 MREs in comparison to the number of miR-195 MREs. Arrows mark the ten genes which contain at least five MREs for both miR-15 family and miR-29 family. The column *fold change* shows whether statistically significant differential expression (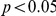) was identified for the gene in our microarray experiments. If so, the fold change and the direction are shown. If not, *n.s.* is shown for not significant.(PDF)

Table S6miR-29 and miR-15 MREs are common in the mRNA sequences of many mammalian species throughout evolution. 7-nucleotide matches for the MREs of miR-29 or miR-15 family miRNAs were counted in the elastin and type I collagen 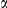 1 and 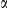 2 genes of five mammalian species. The statistical significance of observing at least as many occurrences of the MRE was estimated using the Poisson distribution (see Methods and Fig. 6 of the main manuscript).(PDF)
