# Supplementary material for: MicroRNAs Differentially Expressed in Postnatal Aortic Development Downregulate Elastin via 3′ UTR and Coding-Sequence Binding Sites
Source: PLoS One. 2011 Jan 31;6(1):e16250. doi: 10.1371/journal.pone.0016250 (PMC3031556; doi:10.1371/journal.pone.0016250)
Supplement: Figure S1 — Validation of differential expression of selected miRNAs (A) and mRNAs (B) by qPCR. The fold change of expression in the aortic samples of six-week old mice compared to that of neonatal mice is shown. Data are presented as mean SEM (). Data was normalized to one RNA sample of the neonatal mice, i.e. 1.0. The mean expression of U6 RNA and snoRNA202 served as endogenous control in microRNA analyses, and Actb as control for gene expression analyses. , . (PDF) [file pone.0016250.s001.pdf]

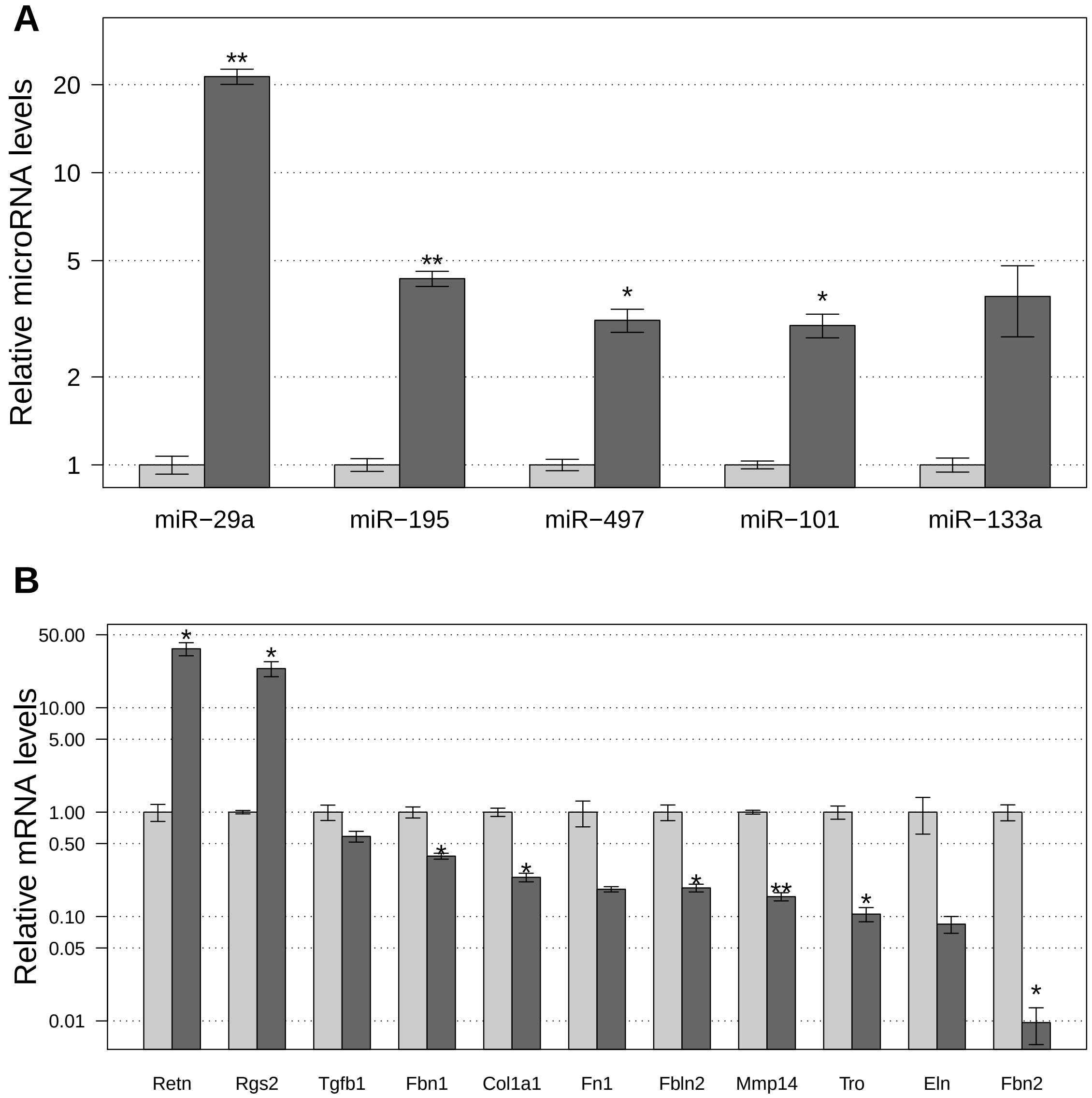


Figure **S1**: Validation of differential expression of selected miRNAs (A) and mRNAs (B) by qPCR. The fold change of expression in the aortic samples of six-week old mice compared to that of neonatal mice is shown. Data are presented as mean ± SEM ($n = 3$). Data was normalized to one RNA sample of the neonatal mice, i.e. 1.0. The mean expression of U6 RNA and snoRNA202 served as endogenous control in microRNA analyses, and *Actb* as control for gene expression analyses. $*p < 0.05$, $**p < 0.01$.
