# Supplementary material for: MicroRNAs Differentially Expressed in Postnatal Aortic Development Downregulate Elastin via 3′ UTR and Coding-Sequence Binding Sites
Source: PLoS One. 2011 Jan 31;6(1):e16250. doi: 10.1371/journal.pone.0016250 (PMC3031556; doi:10.1371/journal.pone.0016250)
Supplement: Figure S2 — Full scans of images of the Western blots presented in the paper (Fig. 1C). The lower panel shows an enhanced exposure of the upper image. (PDF) [file pone.0016250.s002.pdf]

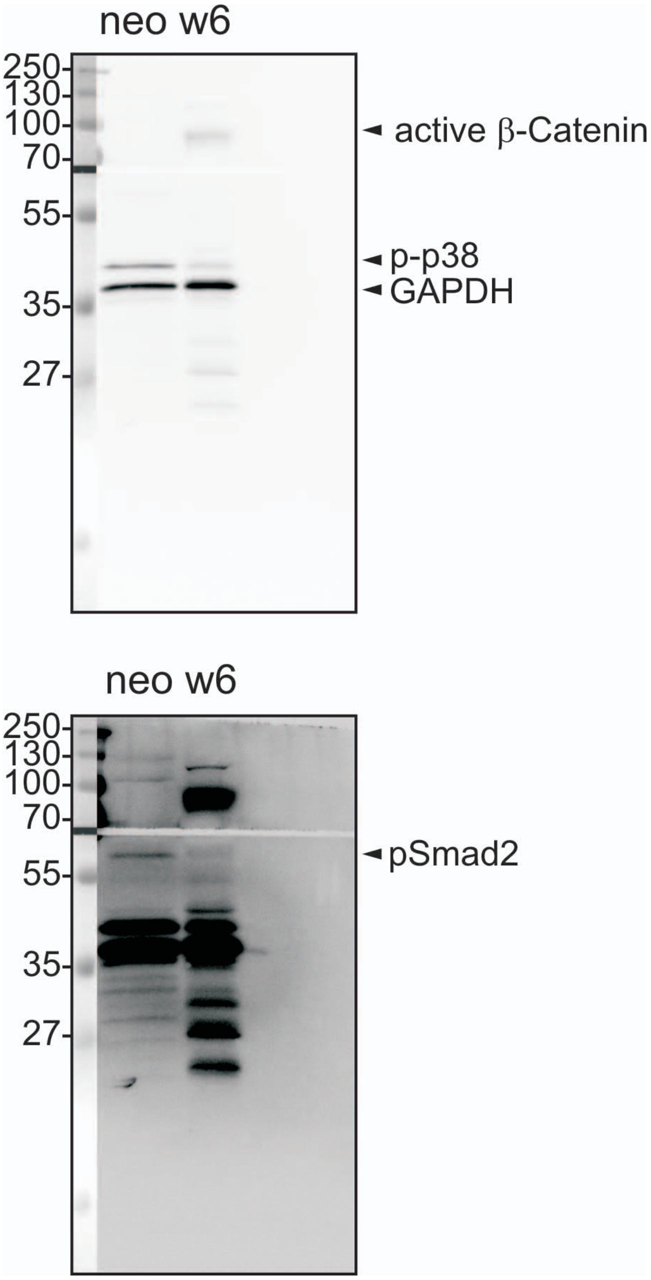

Figure **S2**: Full scans of images of the Western blots presented in the paper (Fig. 1C). The lower panel shows an enhanced exposure of the upper image.
