# Supplementary material for: MicroRNAs Differentially Expressed in Postnatal Aortic Development Downregulate Elastin via 3′ UTR and Coding-Sequence Binding Sites
Source: PLoS One. 2011 Jan 31;6(1):e16250. doi: 10.1371/journal.pone.0016250 (PMC3031556; doi:10.1371/journal.pone.0016250)
Supplement: Figure S3 — Treatment with Anti-miR inhibitor against miR-29a can enhance the expression level of ECM genes in RFL-6 cell culture. Q-PCR for Col1a1, Col1a2, and Eln following treatment of RFL-6 cells with anti-miR-29 (dark grey) compared to untreated control (black). Error bars indicate standard error of triplicate qPCR analysis. The mean expression of Actb and Gapdh served as endogenous control. (PDF) [file pone.0016250.s003.pdf]

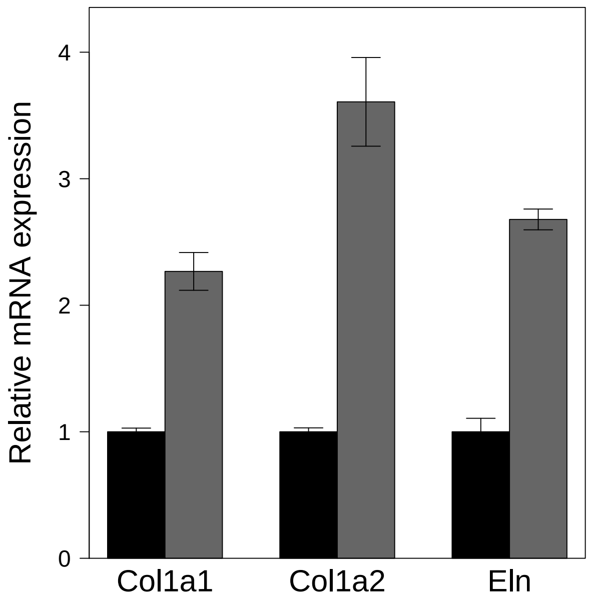

Figure **S3**: Treatment with Anti-miR inhibitor against miR-29a can enhance the expression level of ECM genes in RFL-6 cell culture. Q-PCR for *Col1a1*, *Col1a2*, and *Eln* following treatment of RFL-6 cells with anti-miR-29 (dark grey) compared to untreated control (black). Error bars indicate standard error of triplicate qPCR analysis. The mean expression of *Actb* and *Gapdh* served as endogenous control.
