# Supplementary material for: MicroRNAs Differentially Expressed in Postnatal Aortic Development Downregulate Elastin via 3′ UTR and Coding-Sequence Binding Sites
Source: PLoS One. 2011 Jan 31;6(1):e16250. doi: 10.1371/journal.pone.0016250 (PMC3031556; doi:10.1371/journal.pone.0016250)
Supplement: Table S1 — microRNAs with significantly higher expression in aortic samples from neonatal mice. Data were normalized by setting the threshold of all values to 1. The median shift was normalized to the 75 percentile, and the baseline was transformed using the median of all samples. A subset of genes for data interrogation was generated that excluded probes that were absent or marginal in all of the six samples. Relative expression of each probe in aortic samples of newborn versus six-week old mice was determined. A t-test was performed followed by Benjamini and Hochberg (BH) multiple-testing correction[68]. There were 11 microRNAs with BH-corrected values , foldchange , and normalized mean intensity . An additional six microRNAs were significant at . (PDF) [file pone.0016250.s004.pdf]

Systematic Name	fold change	p-value	corr. p-value (BH)
mmu-miR-410	7.45	1.02×10^{-4}	2.13×10^{-3}
mmu-miR-335-5p	6.15	2.23×10^{-4}	2.43×10^{-3}
mmu-miR-376a	5.12	1.88×10^{-4}	2.13×10^{-3}
mmu-miR-450a-5p	4.85	6.51×10^{-4}	3.54×10^{-3}
mmu-miR-434-3p	4.16	7.91×10^{-4}	3.84×10^{-3}
mmu-miR-377	3.73	1.61×10^{-3}	5.73×10^{-3}
mmu-miR-379	3.51	1.28×10^{-3}	4.86×10^{-3}
mmu-miR-300	3.45	5.23×10^{-4}	3.23×10^{-3}
mmu-miR-411	3.44	1.92×10^{-3}	6.19×10^{-3}
mmu-miR-329	2.95	2.64×10^{-3}	7.19×10^{-3}
mmu-miR-127	2.70	1.92×10^{-3}	6.19×10^{-3}

Table **S1**: microRNAs with significantly higher expression in aortic samples from neonatal mice. Data were normalized by setting the threshold of all values to 1. The median shift was normalized to the 75 percentile, and the baseline was transformed using the median of all samples. A subset of genes for data interrogation was generated that excluded probes that were absent or marginal in all of the six samples. Relative expression of each probe in aortic samples of newborn versus six-week old mice was determined. A *t*-test was performed followed by Benjamini and Hochberg (BH) multiple-testing correction. There were 11 microRNAs with BH-corrected values $p < 0.01$, foldchange > 2, and normalized mean intensity > -1. An additional six microRNAs were significant at $p < 0.05$.
