# Supplementary material for: MicroRNAs Differentially Expressed in Postnatal Aortic Development Downregulate Elastin via 3′ UTR and Coding-Sequence Binding Sites
Source: PLoS One. 2011 Jan 31;6(1):e16250. doi: 10.1371/journal.pone.0016250 (PMC3031556; doi:10.1371/journal.pone.0016250)
Supplement: Table S2 — microRNAs with significantly higher expression in aortic samples from six-week old mice. Data analysis was performed as in Tab. S1. There were 54 microRNAs with BH-corrected values , foldchange , and normalized mean intensity . An additional 17 microRNAs were significant at . (PDF) [file pone.0016250.s005.pdf]

Systematic Name	fold change	p-value	corr. p-value (BH)
mmu-miR-29a	63.23	3.90×10^{-5}	1.12×10^{-3}
mmu-miR-29b	48.62	1.16×10^{-5}	8.67×10^{-4}
mmu-miR-29c	21.48	6.23×10^{-5}	1.52×10^{-3}
mmu-miR-378	16.71	4.09×10^{-4}	3.16×10^{-3}
mmu-miR-195	10.65	1.55×10^{-4}	2.13×10^{-3}
mmu-miR-497	9.73	2.49×10^{-4}	2.49×10^{-3}
mmu-miR-101a	7.06	1.77×10^{-4}	2.13×10^{-3}
mmu-miR-22	6.24	5.17×10^{-4}	3.23×10^{-3}
mmu-miR-1	6.08	2.54×10^{-3}	7.11×10^{-3}
mmu-miR-133a	5.91	3.54×10^{-3}	8.86×10^{-3}
mmu-miR-34a	5.52	1.37×10^{-5}	8.67×10^{-4}
mmu-miR-30e*	5.47	7.38×10^{-4}	3.76×10^{-3}
mmu-miR-30e	5.37	3.98×10^{-4}	3.16×10^{-3}
mmu-miR-193	5.14	2.78×10^{-3}	7.32×10^{-3}
mmu-miR-142-5p	5.04	1.85×10^{-4}	2.13×10^{-3}
mmu-miR-23b	4.99	1.45×10^{-4}	2.13×10^{-3}
mmu-miR-133b	4.91	3.78×10^{-3}	9.32×10^{-3}
mmu-let-7b	4.71	8.63×10^{-4}	3.84×10^{-3}
mmu-miR-23a	4.68	8.27×10^{-4}	3.84×10^{-3}
mmu-miR-30c	4.66	7.79×10^{-4}	3.84×10^{-3}
mmu-miR-30b	4.49	6.14×10^{-4}	3.46×10^{-3}
mmu-let-7c	4.36	8.32×10^{-4}	3.84×10^{-3}
mmu-miR-24-2*	4.31	3.62×10^{-4}	3.01×10^{-3}
mmu-miR-142-3p	4.22	1.70×10^{-4}	2.13×10^{-3}
mmu-miR-145	4.18	1.81×10^{-3}	6.14×10^{-3}
mmu-miR-486	4.17	8.62×10^{-4}	3.84×10^{-3}
mmu-miR-15a	4.16	8.74×10^{-4}	3.84×10^{-3}
mmu-miR-24	4.15	4.64×10^{-4}	3.19×10^{-3}
mmu-miR-144	4.05	1.07×10^{-3}	4.44×10^{-3}
mmu-miR-30a*	3.99	9.90×10^{-4}	4.23×10^{-3}
mmu-miR-27b	3.92	6.63×10^{-4}	3.55×10^{-3}
mmu-miR-143	3.92	3.44×10^{-3}	8.76×10^{-3}
mmu-miR-126-3p	3.81	1.90×10^{-3}	6.19×10^{-3}
mmu-miR-27a	3.70	1.14×10^{-3}	4.61×10^{-3}
mmu-miR-100	3.66	1.53×10^{-3}	5.56×10^{-3}
mmu-miR-101b	3.62	4.43×10^{-4}	3.16×10^{-3}
mmu-miR-30a	3.60	1.12×10^{-3}	4.59×10^{-3}
mmu-miR-30d	3.58	4.85×10^{-4}	3.19×10^{-3}
mcmv-miR-m88-1	3.43	1.24×10^{-3}	4.83×10^{-3}
mmu-let-7g	3.30	2.39×10^{-3}	6.98×10^{-3}
mmu-miR-126-5p	3.18	1.96×10^{-3}	6.19×10^{-3}
mmu-miR-223	3.03	1.95×10^{-3}	6.19×10^{-3}
mmu-miR-99a	3.02	5.29×10^{-4}	3.23×10^{-3}
mmu-let-7f	3.02	2.24×10^{-3}	6.75×10^{-3}
mmu-let-7a	2.97	2.66×10^{-3}	7.19×10^{-3}
mmu-miR-151-5p	2.88	2.59×10^{-3}	7.18×10^{-3}
mmu-miR-720	2.88	2.28×10^{-3}	6.79×10^{-3}
mmu-miR-26b	2.88	3.08×10^{-3}	7.98×10^{-3}
mmu-miR-26a	2.85	2.66×10^{-3}	7.19×10^{-3}
mmu-miR-125b-5p	2.80	1.53×10^{-3}	5.56×10^{-3}
mmu-miR-125a-3p	2.32	2.42×10^{-3}	7.00×10^{-3}
mmu-miR-1897-5p	2.14	1.63×10^{-4}	2.13×10^{-3}
mmu-miR-1895	2.09	1.36×10^{-4}	2.13×10^{-3}
mmu-miR-221	2.07	2.31×10^{-4}	2.43×10^{-3}

Table **S2**: microRNAs with significantly higher expression in aortic samples from six-week old mice. Data analysis was performed as in Tab. S1. There were 54 microRNAs with BH-corrected values $p < 0.01$, foldchange > 2, and normalized mean intensity > -1. An additional 17 microRNAs were significant at $p < 0.05$.
