# Supplementary material for: MicroRNAs Differentially Expressed in Postnatal Aortic Development Downregulate Elastin via 3′ UTR and Coding-Sequence Binding Sites
Source: PLoS One. 2011 Jan 31;6(1):e16250. doi: 10.1371/journal.pone.0016250 (PMC3031556; doi:10.1371/journal.pone.0016250)
Supplement: Table S3 — Genes with significantly higher expression in aortic samples from neonatal mice. Data were normalized by setting the threshold of all values to 1. The median shift was normalized to the 75 percentile, and the baseline was transformed using the median of all samples. A subset of genes for data interrogation was generated that excluded probes that were absent or marginal in all of the six samples. Relative expression of each probe in aortic samples of newborn versus six-week old mice was determined. A t-test was performed followed by Benjamini and Hochberg multiple-testing correction[68]. There were 82 probes corresponding to 78 genes with BH-corrected values and foldchange . An additional 2,064 genes were significant at (Thus, there were a total of 2,338 probes and a total of 2,142 distinct significantly downregulated genes). Genes annotated to the GO term extracellular matrix are shown in bold. (PDF) [file pone.0016250.s006.pdf]

| Probe Name | Gene Symbol | fold change | $p$-value | corr. $p$-value (BH) |
|---|---|---|---|---|
| A_52_P177324 | Dlk1 | 360.70 | $3.53 \times 10^{-5}$ | 0.00983 |
| A_51_P103850 | **Fbn2** | 55.11 | $6.03 \times 10^{-6}$ | 0.00811 |
| A_51_P420600 | Prss35 | 41.32 | $1.09 \times 10^{-5}$ | 0.00857 |
| A_52_P571350 | H19 | 28.63 | $8.69 \times 10^{-6}$ | 0.00835 |
| A_52_P955617 | AK048398 | 26.41 | $2.72 \times 10^{-5}$ | 0.00983 |
| A_52_P1180933 | AK076536 | 22.90 | $1.08 \times 10^{-5}$ | 0.00857 |
| A_52_P508974 | Igf2bp3 | 19.64 | $2.88 \times 10^{-6}$ | 0.00739 |
| A_52_P149017 | 4933426K21Rik | 17.53 | $2.86 \times 10^{-6}$ | 0.00739 |
| A_51_P302336 | Igf2bp3 | 17.33 | $8.10 \times 10^{-6}$ | 0.00820 |
| A_51_P212012 | 8430419K02Rik | 16.26 | $1.84 \times 10^{-6}$ | 0.00739 |
| A_51_P433237 | Cdh3 | 15.70 | $2.33 \times 10^{-5}$ | 0.00961 |
| A_52_P847000 | **Tro** | 15.08 | $1.12 \times 10^{-5}$ | 0.00858 |
| A_51_P302453 | Lhfpl2 | 14.04 | $1.86 \times 10^{-5}$ | 0.00961 |
| A_51_P401907 | EG433016 | 13.06 | $2.29 \times 10^{-5}$ | 0.00961 |
| A_52_P145415 | Ptch2 | 12.18 | $2.34 \times 10^{-5}$ | 0.00961 |
| A_51_P279505 | Rybp | 11.64 | $2.93 \times 10^{-5}$ | 0.00983 |
| A_52_P398925 | Stfa2l1 | 11.53 | $7.85 \times 10^{-6}$ | 0.00815 |
| A_51_P135416 | Mpped2 | 10.68 | $4.43 \times 10^{-7}$ | 0.00372 |
| A_52_P609972 | **Eln** | 10.34 | $7.20 \times 10^{-6}$ | 0.00811 |
| A_51_P409825 | Fndc4 | 10.06 | $1.49 \times 10^{-5}$ | 0.00920 |
| A_52_P384822 | Taok2 | 9.99 | $1.17 \times 10^{-5}$ | 0.00858 |
| A_51_P433141 | **Tro** | 9.25 | $2.66 \times 10^{-6}$ | 0.00739 |
| A_51_P144014 | Gdap5 | 9.24 | $1.37 \times 10^{-5}$ | 0.00907 |
| A_51_P216303 | **Mmp14** | 9.00 | $7.21 \times 10^{-6}$ | 0.00811 |
| A_52_P299974 | **Fn1** | 8.99 | $2.42 \times 10^{-5}$ | 0.00974 |
| A_52_P1107500 | E130118H10Rik | 8.89 | $3.60 \times 10^{-5}$ | 0.00983 |
| A_51_P155482 | Pole | 8.53 | $2.99 \times 10^{-5}$ | 0.00983 |
| A_51_P100856 | **Fn1** | 8.07 | $7.01 \times 10^{-7}$ | 0.00449 |
| A_51_P432432 | Pcdh9 | 8.04 | $3.36 \times 10^{-5}$ | 0.00983 |
| A_52_P475805 | **Col12a1** | 7.50 | $1.34 \times 10^{-5}$ | 0.00907 |
| A_51_P216965 | Fkbp10 | 7.36 | $2.58 \times 10^{-5}$ | 0.00983 |
| A_51_P306689 | Rcor2 | 7.32 | $3.64 \times 10^{-5}$ | 0.00983 |
| A_51_P461005 | Nrk | 7.21 | $2.04 \times 10^{-5}$ | 0.00961 |
| A_51_P471458 | Sult5a1 | 6.64 | $1.03 \times 10^{-5}$ | 0.00841 |
| A_51_P277629 | Zfp81 | 6.51 | $1.02 \times 10^{-5}$ | 0.00841 |
| A_52_P590535 | **Fbln2** | 6.47 | $1.01 \times 10^{-5}$ | 0.00841 |
| A_52_P569178 | Sf3a1 | 6.33 | $1.75 \times 10^{-5}$ | 0.00961 |
| A_52_P623457 | Hic2 | 6.20 | $1.51 \times 10^{-5}$ | 0.00920 |
| A_51_P351970 | Hells | 6.05 | $5.40 \times 10^{-6}$ | 0.00811 |
| A_51_P273556 | 2310007D09Rik | 6.01 | $3.03 \times 10^{-5}$ | 0.00983 |
| A_51_P511448 | Rutbc2 | 5.97 | $3.72 \times 10^{-5}$ | 0.00983 |
| A_52_P1067 | F830028O17Rik | 5.95 | $3.10 \times 10^{-5}$ | 0.00983 |
| A_52_P653456 | TC1680672 | 5.79 | $2.28 \times 10^{-5}$ | 0.00961 |
| A_52_P557156 | Tmtc2 | 5.55 | $3.76 \times 10^{-5}$ | 0.00983 |
| A_51_P448545 | 2810406K13Rik | 5.44 | $3.15 \times 10^{-5}$ | 0.00983 |
| A_52_P1037117 | BB078705 | 5.24 | $3.75 \times 10^{-5}$ | 0.00983 |
| A_52_P344036 | **Adamts7** | 5.20 | $2.27 \times 10^{-5}$ | 0.00961 |
| A_51_P155582 | Rad51ap1 | 4.88 | $2.31 \times 10^{-5}$ | 0.00961 |
| A_51_P227866 | Txndc13 | 4.74 | $2.65 \times 10^{-5}$ | 0.00983 |
| A_51_P500474 | AK078994 | 4.56 | $3.57 \times 10^{-5}$ | 0.00983 |
| A_52_P223495 | Itga4 | 4.54 | $3.58 \times 10^{-5}$ | 0.00983 |
| A_51_P249777 | Vangl2 | 4.54 | $3.87 \times 10^{-5}$ | 0.00996 |
| A_51_P333279 | Hcn3 | 4.30 | $6.88 \times 10^{-6}$ | 0.00811 |
| A_51_P131494 | Foxk2 | 4.17 | $2.26 \times 10^{-5}$ | 0.00961 |
| A_52_P664405 | Zfp286 | 4.07 | $2.07 \times 10^{-5}$ | 0.00961 |
| A_52_P286342 | Dhx33 | 4.02 | $1.37 \times 10^{-5}$ | 0.00907 |
| A_51_P317836 | Cetn1 | 3.94 | $1.84 \times 10^{-5}$ | 0.00961 |
| A_52_P255458 | Air | 3.93 | $9.51 \times 10^{-6}$ | 0.00841 |
| A_51_P313093 | Ick | 3.88 | $3.09 \times 10^{-5}$ | 0.00983 |
| A_51_P189722 | Cabin1 | 3.88 | $3.45 \times 10^{-7}$ | 0.00372 |
| A_52_P645357 | Adipor1 | 3.85 | $4.36 \times 10^{-7}$ | 0.00372 |
| A_51_P199987 | Gucy1a3 | 3.80 | $3.08 \times 10^{-5}$ | 0.00983 |
| A_51_P285779 | Asphd2 | 3.78 | $9.54 \times 10^{-6}$ | 0.00841 |
| A_52_P59264 | Sncaip | 3.47 | $1.55 \times 10^{-5}$ | 0.00920 |
| A_51_P485828 | Arid3a | 3.44 | $3.70 \times 10^{-5}$ | 0.00983 |
| A_51_P370678 | Gfi1b | 3.38 | $2.50 \times 10^{-6}$ | 0.00739 |
| A_52_P496341 | Cic | 3.22 | $4.84 \times 10^{-7}$ | 0.00372 |
| A_52_P319495 | Boc | 3.21 | $3.25 \times 10^{-6}$ | 0.00778 |
| A_52_P663789 | Rcor1 | 3.14 | $3.52 \times 10^{-5}$ | 0.00983 |
| A_51_P212741 | Scn2b | 3.12 | $1.55 \times 10^{-5}$ | 0.00920 |
| A_52_P131812 | 6720469N11Rik | 3.05 | $1.75 \times 10^{-5}$ | 0.00961 |
| A_52_P525107 | **Col1a1** | 2.94 | $1.23 \times 10^{-5}$ | 0.00870 |

*Continued on Next Page…*

Table **S2** – Continued

| Probe Name | Gene Symbol | fold change | $p$-value | corr. $p$-value (BH) |
|---|---|---|---|---|
| A_51_P178022 | Plekhg2 | 2.76 | $2.70 \times 10^{-5}$ | 0.00983 |
| A_51_P397639 | Trrap | 2.71 | $1.17 \times 10^{-5}$ | 0.00858 |
| A_52_P441974 | Evc2 | 2.65 | $3.70 \times 10^{-5}$ | 0.00983 |
| A_51_P105241 | 4931428F04Rik | 2.59 | $2.55 \times 10^{-5}$ | 0.00983 |
| A_51_P485220 | Csnk1g1 | 2.51 | $1.54 \times 10^{-5}$ | 0.00920 |
| A_52_P289256 | Arid1b | 2.50 | $3.66 \times 10^{-5}$ | 0.00983 |
| A_52_P422859 | M77174 | 2.37 | $2.32 \times 10^{-5}$ | 0.00961 |
| A_52_P1067916 | AK043132 | 2.24 | $1.02 \times 10^{-5}$ | 0.00841 |
| A_52_P82532 | Bcl7c | 2.23 | $3.11 \times 10^{-5}$ | 0.00983 |

Table **S3**: Genes with significantly higher expression in aortic samples from neonatal mice. Data were normalized by setting the threshold of all values to 1. The median shift was normalized to the 75 percentile, and the baseline was transformed using the median of all samples. A subset of genes for data interrogation was generated that excluded probes that were absent or marginal in all of the six samples. Relative expression of each probe in aortic samples of newborn versus six-week old mice was determined. A *t*-test was performed followed by Benjamini and Hochberg multiple-testing correction[**?**]. There were 82 probes corresponding to 78 genes with BH-corrected values $p < 0.01$ and foldchange $> 2$. An additional 2,064 genes were significant at $p < 0.05$ (Thus, there were a total of 2,338 probes and a total of 2,142 distinct significantly downregulated genes). Genes annotated to the GO term *extracellular matrix* are shown in bold.
