# Supplementary material for: MicroRNAs Differentially Expressed in Postnatal Aortic Development Downregulate Elastin via 3′ UTR and Coding-Sequence Binding Sites
Source: PLoS One. 2011 Jan 31;6(1):e16250. doi: 10.1371/journal.pone.0016250 (PMC3031556; doi:10.1371/journal.pone.0016250)
Supplement: Table S4 — Genes with significantly higher expression in aortic samples from six-week old mice. Data analysis was performed as in Tab. S3. There were 57 probes corresponding to 56 genes with BH-corrected values and foldchange . An additional 1,271 genes were significant at (Thus, there were a total of 1,509 probes and 1,327 sigificantly upregulated genes). Genes annotated to the GO term mitochondrion are shown in bold. (PDF) [file pone.0016250.s007.pdf]

| Probe Name | Gene Symbol | fold change | $p$-value | corr. $p$-value (BH) |
|---|---|---|---|---|
| A_51_P375969 | Ces3 | 92.33 | $1.75 \times 10^{-5}$ | 0.00961 |
| A_51_P178772 | AU018778 | 78.10 | $2.00 \times 10^{-5}$ | 0.00961 |
| A_51_P336070 | Lctl | 60.75 | $2.18 \times 10^{-5}$ | 0.00961 |
| A_52_P154971 | **Slc27a2** | 37.47 | $6.81 \times 10^{-6}$ | 0.00811 |
| A_51_P321126 | Fasn | 35.74 | $5.37 \times 10^{-6}$ | 0.00811 |
| A_52_P55972 | Retn | 34.71 | $3.04 \times 10^{-5}$ | 0.00983 |
| A_52_P318040 | Acaca | 21.94 | $2.48 \times 10^{-5}$ | 0.00974 |
| A_51_P114094 | Clstn3 | 21.33 | $4.82 \times 10^{-6}$ | 0.00811 |
| A_52_P90363 | Ifi27 | 19.83 | $7.54 \times 10^{-6}$ | 0.00811 |
| A_51_P233597 | Retn | 18.95 | $7.59 \times 10^{-6}$ | 0.00811 |
| A_52_P378968 | Rgs2 | 17.34 | $1.18 \times 10^{-5}$ | 0.00858 |
| A_51_P263591 | Pank1 | 16.63 | $2.86 \times 10^{-6}$ | 0.00739 |
| A_51_P444301 | **Brp44l** | 13.70 | $1.99 \times 10^{-5}$ | 0.00961 |
| A_51_P465292 | Hnmt | 13.53 | $3.14 \times 10^{-5}$ | 0.00983 |
| A_51_P384936 | Ddo | 11.70 | $2.95 \times 10^{-5}$ | 0.00983 |
| A_51_P420547 | Clic5 | 11.07 | $5.07 \times 10^{-6}$ | 0.00811 |
| A_51_P292550 | **Chdh** | 8.94 | $3.32 \times 10^{-5}$ | 0.00983 |
| A_51_P479321 | **Acss1** | 7.72 | $3.38 \times 10^{-5}$ | 0.00983 |
| A_51_P197528 | Ly6c | 7.64 | $1.87 \times 10^{-5}$ | 0.00961 |
| A_51_P517430 | Cd1d1 | 7.32 | $2.68 \times 10^{-5}$ | 0.00983 |
| A_52_P200359 | **Aco2** | 7.05 | $2.76 \times 10^{-5}$ | 0.00983 |
| A_52_P251690 | Gvin1 | 6.98 | $2.87 \times 10^{-5}$ | 0.00983 |
| A_52_P680935 | Ghr | 6.88 | $1.43 \times 10^{-5}$ | 0.00920 |
| A_51_P100828 | **Prdx3** | 6.79 | $2.89 \times 10^{-5}$ | 0.00983 |
| A_51_P128929 | **Alas1** | 6.43 | $3.07 \times 10^{-5}$ | 0.00983 |
| A_52_P59681 | Hrsp12 | 5.10 | $3.85 \times 10^{-6}$ | 0.00778 |
| A_51_P155212 | **Etfb** | 5.05 | $3.47 \times 10^{-5}$ | 0.00983 |
| A_51_P365468 | Clpb | 5.05 | $3.03 \times 10^{-5}$ | 0.00983 |
| A_52_P724940 | **Ndufa12** | 4.86 | $3.59 \times 10^{-5}$ | 0.00983 |
| A_51_P304170 | Rtp4 | 4.83 | $5.30 \times 10^{-6}$ | 0.00811 |
| A_51_P273609 | Itpka | 4.58 | $6.70 \times 10^{-6}$ | 0.00811 |
| A_52_P547795 | **Opa1** | 4.52 | $3.52 \times 10^{-6}$ | 0.00778 |
| A_52_P177021 | Pts | 4.51 | $2.11 \times 10^{-6}$ | 0.00739 |
| A_52_P54439 | Nck1 | 4.37 | $3.55 \times 10^{-5}$ | 0.00983 |
| A_52_P582105 | **Fdx1** | 4.07 | $1.80 \times 10^{-5}$ | 0.00961 |
| A_51_P191463 | ENSMUST00000077662 | 3.81 | $3.78 \times 10^{-5}$ | 0.00983 |
| A_51_P190697 | **Mrps36** | 3.77 | $1.80 \times 10^{-5}$ | 0.00961 |
| A_51_P290207 | Insig1 | 3.60 | $1.24 \times 10^{-5}$ | 0.00870 |
| A_52_P680735 | **Aifm1** | 3.55 | $3.53 \times 10^{-5}$ | 0.00983 |
| A_52_P86176 | Tap2 | 3.52 | $8.69 \times 10^{-6}$ | 0.00835 |
| A_51_P207849 | Bola1 | 3.51 | $1.32 \times 10^{-7}$ | 0.00372 |
| A_51_P305583 | Sp100 | 3.37 | $1.61 \times 10^{-5}$ | 0.00937 |
| A_51_P163305 | Nudt5 | 3.26 | $3.80 \times 10^{-6}$ | 0.00778 |
| A_51_P314323 | Lsmd1 | 3.25 | $3.34 \times 10^{-5}$ | 0.00983 |
| A_51_P115159 | 2310056P07Rik | 3.23 | $3.20 \times 10^{-5}$ | 0.00983 |
| A_52_P353322 | Cog4 | 3.14 | $6.01 \times 10^{-6}$ | 0.00811 |
| A_51_P492410 | Pmvk | 3.02 | $2.20 \times 10^{-5}$ | 0.00961 |
| A_52_P460584 | Tnfrsf25 | 2.87 | $3.71 \times 10^{-5}$ | 0.00983 |
| A_51_P143142 | **Mrpl12** | 2.83 | $2.20 \times 10^{-5}$ | 0.00961 |
| A_52_P431872 | **Ptcd3** | 2.60 | $4.30 \times 10^{-6}$ | 0.00811 |
| A_51_P490198 | Pop5 | 2.60 | $2.48 \times 10^{-5}$ | 0.00974 |
| A_52_P423174 | Acaa1b | 2.56 | $2.35 \times 10^{-5}$ | 0.00961 |
| A_52_P570266 | Psmb10 | 2.47 | $3.91 \times 10^{-5}$ | 0.00996 |
| A_52_P617080 | Txndc14 | 2.39 | $1.93 \times 10^{-5}$ | 0.00961 |
| A_52_P34043 | Ppm1b | 2.37 | $1.38 \times 10^{-6}$ | 0.00739 |
| A_52_P443500 | Krcc1 | 2.28 | $2.40 \times 10^{-6}$ | 0.00739 |
| A_51_P136699 | Ttc7b | 2.07 | $9.33 \times 10^{-6}$ | 0.00841 |

Table **S4**: Genes with significantly higher expression in aortic samples from six-week old mice. Data analysis was performed as in Tab. S3. There were 57 probes corresponding to 56 genes with BH-corrected values $p < 0.01$ and foldchange $> 2$. An additional 1,271 genes were significant at $p < 0.05$ (Thus, there were a total of 1,509 probes and 1,327 sigificantly upregulated genes). Genes annotated to the GO term *mitochondrion* are shown in bold.
