# Supplementary material for: MicroRNAs Differentially Expressed in Postnatal Aortic Development Downregulate Elastin via 3′ UTR and Coding-Sequence Binding Sites
Source: PLoS One. 2011 Jan 31;6(1):e16250. doi: 10.1371/journal.pone.0016250 (PMC3031556; doi:10.1371/journal.pone.0016250)
Supplement: Table S5 — There are 50 genes containing at least five MREs for miR-29 in the CDS plus 3′ UTR. MREs were identified using the definitions of 7–8 mer canonical miR seed-matches. As shown in Fig. 3 of the main manuscript, the first nucleotide of the seed sequences of miR-195 and miR-497 is different (). Therefore, some genes have a lower number of miR-497 MREs in comparison to the number of miR-195 MREs. Arrows mark the ten genes which contain at least five MREs for both miR-15 family and miR-29 family. The column fold change shows whether statistically significant differential expression () was identified for the gene in our microarray experiments. If so, the fold change and the direction are shown. If not, n.s. is shown for not significant. (PDF) [file pone.0016250.s008.pdf]

		miR-29abc		miR-195		miR-497		
	gene	CDS	3' UTR	CDS	3' UTR	CDS	3' UTR	fold change
	Abcc10	6	-	3	-	3	-	n.s.
	Adamts17	2	3	4	-	3	-	n.s.
	AI414108	-	6	-	1	-	1	n.s.
	Atad2b	3	3	1	1	1	1	4.4 ↓
	Brwd3	4	1	2	-	2	-	n.s.
	Cacna1b	6	-	3	-	3	-	2.6 ↓
	Cecr2	-	5	1	-	-	-	n.s.
	Col1a1	22	-	3	-	3	-	9.1 ↓
→	Col1a2	18	1	7	-	7	-	3.2 ↓
	Col2a1	13	1	2	-	2	-	2.0 ↓
	Col3a1	12	2	2	-	2	-	n.s.
	Col4a1	3	2	1	-	1	-	3.5 ↓
→	Col5a1	2	4	5	1	5	1	8.7 ↓
	Col5a2	5	2	1	-	1	-	4.7 ↓
	Col5a3	2	3	1	-	1	-	n.s.
	Col7a1	6	2	1	-	1	-	n.s.
	Col11a1	5	2	1	-	1	-	n.s.
	Col22a1	3	3	-	-	-	-	n.s.
	D230004J03Rik	3	10	-	4	-	4	n.s.
	D630045J12Rik	1	4	3	-	3	-	n.s.
→	Dnahc1	7	-	5	-	5	-	n.s.
→	Dnahc17	5	-	5	-	3	-	5.3 ↓
	Dnmt3a	1	6	-	-	-	-	4.0 ↓
	Dot1l	4	2	2	-	2	-	n.s.
→	Eln	11	3	13	-	8	-	10.3 ↓
→	Eppk1	10	-	15	-	14	-	n.s.
	Gabpb2	-	5	1	2	-	2	n.s.
	Gpr156	3	3	3	1	2	1	n.s.
	Gpr98	7	-	4	-	4	-	n.s.
	Hif3a	2	3	-	1	-	1	n.s.
	Itih5l	-	8	-	4	-	2	n.s.
→	Kcnq1ot1	-	10	-	15	-	7	n.s.
	Kdm6b	4	1	1	-	1	-	n.s.
	LOC633371	-	6	-	4	-	2	n.s.
	Loxl2	3	2	-	1	-	1	3.8 ↓
	Nav1	1	4	-	2	-	1	n.s.
	Nbea	5	-	4	-	4	-	5.6 ↓
	Neb	9	-	4	-	2	-	n.s.
	Obsl1	10	-	4	-	3	-	n.s.
→	Pkd1	8	-	6	1	5	1	n.s.
	Plg	5	-	-	-	-	-	n.s.
	Pnpla3	-	5	-	-	-	-	79.0 ↑
	Slc30a10	2	3	-	-	-	-	n.s.
	Tet1	5	8	-	1	-	-	n.s.
	Tet2	2	5	1	-	-	-	n.s.
→	Traf3	1	4	1	5	1	5	n.s.
	Trib2	2	3	1	-	-	-	3.1 ↓
→	Ttn	11	-	17	-	15	-	5.2 ↓
	Zbtb34	1	4	-	3	-	3	n.s.
	Zzef1	4	1	2	-	2	-	3.8 ↓

Table **S5**: There are 50 genes containing at least five MREs for miR-29 in the CDS plus 3' UTR. MREs were identified using the definitions of 7–8 mer canonical miR seed-matches. As shown in Fig. 3 of the main manuscript, the first nucleotide of the seed sequences of miR-195 and miR-497 is different ($U \rightarrow C$). Therefore, some genes have a lower number of miR-497 MREs in comparison to the number of miR-195 MREs. Arrows mark the ten genes which contain at least five MREs for both miR-15 family and miR-29 family. The column *fold change* shows whether statistically significant differential expression ($p < 0.05$) was identified for the gene in our microarray experiments. If so, the fold change and the direction are shown. If not, *n.s.* is shown for not significant.
