# Supplementary material for: MicroRNAs Differentially Expressed in Postnatal Aortic Development Downregulate Elastin via 3′ UTR and Coding-Sequence Binding Sites
Source: PLoS One. 2011 Jan 31;6(1):e16250. doi: 10.1371/journal.pone.0016250 (PMC3031556; doi:10.1371/journal.pone.0016250)
Supplement: Table S6 — miR-29 and miR-15 MREs are common in the mRNA sequences of many mammalian species throughout evolution. 7-nucleotide matches for the MREs of miR-29 or miR-15 family miRNAs were counted in the elastin and type I collagen 1 and 2 genes of five mammalian species. The statistical significance of observing at least as many occurrences of the MRE was estimated using the Poisson distribution (see Methods and Fig. 6 of the main manuscript). (PDF) [file pone.0016250.s009.pdf]

Species	miRNA	Elastin		Collagen, type I, α 1		Collagen, type I, α 2	
		n	p-value	n	p-value	n	p-value
Mouse	miR-29	14	2.37×10^{-21}	21	6.12×10^{-33}	16	5.39×10^{-23}
	miR-15	8	5.97×10^{-11}	3	2.23×10^{-3}	7	1.91×10^{-8}
Rat	miR-29	13	6.52×10^{-20}	21	3.40×10^{-31}	15	2.70×10^{-22}
	miR-15	9	7.37×10^{-13}	5	1.78×10^{-5}	7	6.35×10^{-9}
Human	miR-29	6	6.13×10^{-8}	25	4.1×10^{-38}	18	4.73×10^{-26}
	miR-15	1	0.17	3	4.53×10^{-3}	6	7.49×10^{-7}
Cow	miR-29	3	4.50×10^{-4}	15	3.27×10^{-23}	15	3.27×10^{-23}
	miR-15	6	1.18×10^{-8}	4	7.35×10^{-5}	6	9.49×10^{-8}
Dog	miR-29	5	3.02×10^{-7}	19	2.46×10^{-29}	11	3.54×10^{-15}
	miR-15	4	1.08×10^{-5}	2	2.59×10^{-2}	6	2.14×10^{-7}

Table **S6**: miR-29 and miR-15 MREs are common in the mRNA sequences of many mammalian species throughout evolution. 7-nucleotide matches for the MREs of miR-29 or miR-15 family miRNAs were counted in the elastin and type I collagen α 1 and α 2 genes of five mammalian species. The statistical significance of observing at least as many occurrences of the MRE was estimated using the Poisson distribution (see Methods and Fig. 6 of the main manuscript).
